# Dietary cysteine drives body fat loss via FMRFamide signaling in *Drosophila* and mouse

**DOI:** 10.1038/s41422-023-00800-8

**Published:** 2023-04-13

**Authors:** Tingting Song, Wusa Qin, Zeliang Lai, Haoyu Li, Daihan Li, Baojia Wang, Wuquan Deng, Tingzhang Wang, Liming Wang, Rui Huang

**Affiliations:** 1 Institute of Molecular Physiology, Shenzhen Bay Laboratory, Shenzhen, Guangdong, China; 2 Center for Neurointelligence, School of Medicine, Chongqing University, Chongqing, China; 3 Department of Endocrinology and Nephrology, Chongqing University Central Hospital, Chongqing Emergency Medical Center, Chongqing, China; 4 Department of General Surgery, Sir Run Run Shaw Hospital, Zhejiang University School of Medicine, Hangzhou, Zhejiang, China

**Keywords:** Mechanisms of disease, Hormone receptors

## Abstract

Obesity imposes a global health threat and calls for safe and effective therapeutic options. Here, we found that protein-rich diet significantly reduced body fat storage in fruit flies, which was largely attributed to dietary cysteine intake. Mechanistically, dietary cysteine increased the production of a neuropeptide FMRFamide (FMRFa). Enhanced FMRFa activity simultaneously promoted energy expenditure and suppressed food intake through its cognate receptor (FMRFaR), both contributing to the fat loss effect. In the fat body, FMRFa signaling promoted lipolysis by increasing PKA and lipase activity. In sweet-sensing gustatory neurons, FMRFa signaling suppressed appetitive perception and hence food intake. We also demonstrated that dietary cysteine worked in a similar way in mice via neuropeptide FF (NPFF) signaling, a mammalian RFamide peptide. In addition, dietary cysteine or FMRFa/NPFF administration provided protective effect against metabolic stress in flies and mice without behavioral abnormalities. Therefore, our study reveals a novel target for the development of safe and effective therapies against obesity and related metabolic diseases.

## Introduction

Obesity has multiplied in the past few decades worldwide and will continue to rise in most countries, which leads to huge direct and indirect healthcare costs for more than $700 billion each year.^1^ World Health Organization reported that worldwide 39% of adults were overweight, of whom 13% were obese.^2^ Obesity is characterized by excessive or abnormal body fat deposit which may also lead to chronic inflammatory response.^3^ Obesity-induced inflammation is crucial to the development of insulin resistance, and the latter eventually perturbs metabolic homeostasis and thus contributes to various metabolic diseases, including type 2 diabetes and cardiovascular diseases.^3,4^ Dysregulated adipose tissue also exerts a direct effect on the central nervous system by secreting a series of adipokines, resulting in many mental health issues, such as depression, bipolar disorder, anxiety, and schizophrenia.^5^

Recent guidelines recommend that overweight and obese people aim to lose 5% or more of their body weight to gain significant health improvements.^6^ Even a modest weight loss can generate considerable metabolic benefits, such that the insulin sensitivity of fat, muscle and liver, as well as β cell function can be largely improved. As a result, the risk metabolites for heart disease and diabetes, such as circulatory glucose, triglycerides, insulin, and leptin, can also be significantly decreased.^7^ These benefits may explain the clinical observations of the tight association between weight loss and improvement in obesity-related comorbidities such as hypertension, type 2 diabetes, and non-alcoholic fatty liver disease, as well as improvement in blood glucose and lipid profiles.^8^

Common medical interventions for weight loss include several surgical and pharmacological approaches. Surgery is the only clinical treatment with significant and long-lasting effect, but is merely suitable for severely obese patients.^9^ Pharmacotherapies provide a less-invasive and possibly safer option for those individuals who were not suitable for weight loss surgery, reducing body weight via suppressing nutrient intake and/or promoting energy expenditure.^10^ Among them, glucagon-like peptide 1 (GLP1) analogs or agonists hold promise as a first-line anti-obesity treatment, which functions in a way by delaying gastric emptying, accelerating insulin secretion, and suppressing appetite and food intake.^10,11^ In addition, fibroblast growth factor 21 (FGF21) has been shown to significantly induce weight loss and increase insulin sensitivity without hypoglycemia or decrease in food intake.^12^ Besides, inhibitors of fat/glucose absorption and activators of catabolism may also be viable approaches in weight loss.^10^ Although offering great promise, the side effects of these drugs have limited their clinical application. For instance, current anti-obesity medications targeting food intake usually enhance a series of anxiety-related behaviors and emotionality, while some drugs targeting fat tissue raise the risk of cardiovascular diseases.^13,14^

Besides these medical interventions, there are also strategies for weight management that mainly focus on lifestyle changes. Behavioral interventions via diet change and regular exercise are reported to induce significant weight loss in the short term, but hardly avoid weight regain in the long term.^15^ Meanwhile, severe dietary restriction has lots of negative health consequences, such as malnutrition, disordered food intake, and emotional distress.^16^ Dietary modulations including ketogenic diet (a low-carb, high-fat diet) and high-protein diet (HPD) have also been shown to be effective weight managing approaches. Clinical reports have found that HPD effectively reduces body weight and also prevents weight regain,^17^ and decrease in food intake is a major underlying mechanism. HPD increases satiety via the release of a group of anorexigenic hormones such as cholecystokinin (CCK), GLP-1, and peptide tyrosine-tyrosine.^17,18^ Conversely, ghrelin, an orexigenic hormone that promotes food intake, is suppressed by HPD.^19^ Attention should be paid to patients’ renal function, however, because of a large quantity of amino acid metabolites.^20^ To optimize the health benefit of HPD, it is essential to uncover the detailed weight-modulating mechanism of HPD and its exact functioning component.

Fruit fly *Drosophila melanogaster* offers a simple and powerful model to study the regulation and dysregulation of organismal metabolism, since the overall logic and key components of neuronal and metabolic regulatory pathways are largely conserved in flies.^21–23^ The major metabolic organs and key metabolites are comparable between mammals and flies. For example, the fat body of flies serves as a major tissue for energy storage and mobilization that is analogous to mammalian adipose tissue and liver.^24^ The peripheral chemosensory systems of fruit flies including the olfactory and gustatory systems have been shown to regulate the search, localization, assessment, and acquisition of desirable food sources in a way similar to those in mammals.^25–27^ The central nervous system of fruit flies senses and integrates the nutrient needs and regulates foraging and feeding behavior accordingly with insulin signaling as a core component.^24,28^ Last but not least, a great deal of metabolic disease-associated genes are highly conserved between flies and human,^29^ and the pathogenesis of obesity and type 2 diabetes in flies is also similar to human.^30^

Many conserved metabolic mechanisms have been identified in flies.^31,32^ Amongst these complex regulatory mechanisms, neuropeptidergic systems play a central role in the neural regulation of metabolism and behavior in response to environmental cues and internal nutrient needs.^28,31^ Insulin-producing cells (IPCs) and adipokinetic hormone (AKH)-producing cells, which produce *Drosophila* insulin-like peptides (DILPs) and AKH, respectively, play a central role in detecting internal nutrient status and maintaining glucose and lipid homeostasis.^24^ As a hunger signal like mammalian glucagon, AKH increases circulating trehalose by stimulating lipolysis, and triggers food-seeking behavior.^28,33^ DILPs are the fly equivalents of mammalian insulin, functioning as satiety signals to activate lipogenesis and suppress feeding behavior.^34,35^ Besides DILPs and AKH, various neuropeptidergic systems are involved in the maintenance of amino acid homeostasis, including Diuretic hormone 44 (DH44), hugin, and FIT.^36–38^ Meanwhile, neuropeptides are involved in the fine-tuning of various aspects of feeding behaviors. SIFamide and short neuropeptide F (sNPF) enhance appetitive odor-guided behavior, facilitating food localization.^25,39^ Myoinhibitory peptides, Allatostatin A, and Neuropeptide F modulate gustatory sensitivity to food cues.^27,28,40,41^ DH44, hugin, tackykinin, Corazonin, drosulfakinin, and FIT have all been shown to modulate the amount of food consumption.^35,36,38,40^ These neuropeptidergic signals may be further integrated by the IPCs to generate desirable and appropriate behavioral output.^35,42,43^

In this study, we first confirmed the fat loss effect of HPD in fruit flies *Drosophila melanogaster*, and further investigated whether and how it modulated food intake and energy expenditure. We found that one single component in HPD, cysteine, was sufficient to recapitulate the effect of HPD, via both suppressing food intake and promoting lipid degradation. Via several tissue-specific RNAi screens, we identified that a neuropeptide FMRFamide (FMRFa) and its cognate receptor FMRFaR together mediated the fat loss effect of dietary cysteine. Upon dietary cysteine intake, enhanced FMRFa signaling exerted dual effects: in the fat body, FMRFa directly triggered lipid degradation via activating FMRFaR and the downstream PKA pathway; in the nervous system, FMRFa reduced the sensitivity of sweet-sensing gustatory neurons and hence food consumption, also via FMRFaR. Meanwhile, dietary cysteine did not affect flies’ overall survival and behavioral output, but offered significant protection against the metabolic dysfunctions induced by high-fat diet (HFD). We also found that in mice, dietary cysteine feeding also exhibited conserved metabolic and behavioral effect via neuropeptide FF (NPFF), a mammalian RFamide peptide. Taken together, our study discovered dietary cysteine supplement as a novel and effective strategy for weight management with no obvious adverse effect and elucidated its underlying mechanism. It might contribute to the worldwide effort to mitigate obesity and related metabolic disorders.

## Results

### Dietary proteins reduced body fat by increasing energy expenditure and suppressing food intake

**Fig. 1:**
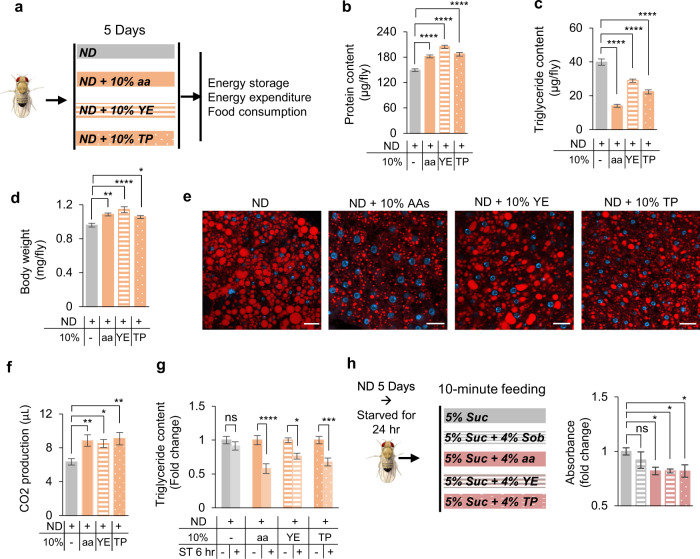
HPD decreased fat mass by increasing lipid degradation and suppressing food intake. **a** Illustration of experimental design in **b**–**g**. Wild-type *Oregon-R* virgin female flies were collected and fed with ND (grey) or HPD (ND + 10% indicated HPD, orange) for 5 days and then subjected to different metabolic and behavioral assays. **b**–**d** Protein content (**b**, *n* = 10), triglyceride content (**c**, *n* = 8), and body weight (**d**, *n* = 7–10) of flies after treatment with different types of HPD as indicated. **e** Nile Red staining of the fat bodies collected from the indicated treatment groups. Lipids are in red and nucleus in blue. Scale bars, 20 μm. **f** CO_2_ production of the indicated treatment groups within 1 h (*n* = 11–16 biological replicates, each containing 5 flies). **g** Triglyceride content of flies pre-fed with different types of HPD for 5 days and then starved for 6 h or not (*n* = 8). **h** Food consumed in a 10-min episode. Flies were fed with ND for 5 days and then starved for 24 h before the assay (*n* = 6–13). ns, *P* > 0.05; *P < 0.05; **P < 0.01; ***P < 0.001;  ****P < 0.0001. One-way ANOVA followed by post hoc test with Bonferroni correction was used for multiple comparisons when applicable.

Protein-rich diet has been shown to reduce body fat deposit while increasing muscle mass.^44^ We first explored the effect of HPD on overall metabolism in *Drosophila* (Fig. 1a). Three types of HPD we tested (standard fly medium (termed ND, normal diet) supplemented with 10% amino acid mixture, yeast extract, or tryptone) significantly increased the protein content and reduced the lipid storage of flies, whereas the body weight was slightly increased (Fig. 1b–d). Within the fat body, the main site of lipid storage of fruit flies, the size of intracellular lipid droplets was also significantly reduced after HPD feeding (Fig. 1e). These results suggest that HPD feeding led to a decrease of body fat deposit.

We further examined how HPD feeding decreased body fat. One possible explanation was that HPD enhanced energy expenditure. Indeed, flies fed with HPD exhibited increased CO_2_ production compared to flies fed with ND (Fig. 1f). Consistently, upon 6-h food deprivation, flies previously fed with HPD exhibited faster reduction of body fat storage (Fig. 1g).

Another possibility was that HPD suppressed feeding behavior and hence energy intake. In line with this possibility, we found that starved flies consumed significantly less food when fed with HPD (Fig. 1h), which was consistent with a previous report showing the presence of “satiety signal” in dietary proteins.^38,45^ The “satiety signal” of HPD seemed not due to increased energy content in HPD, since addition of sorbitol (a nutritive but tasteless sugar) did not affect acute food consumption (Fig. 1h).

To further verify the effect of HPD feeding, we adopted a minimal food medium (5% sucrose) and found that adding protein sources in it also induced energy expenditure and reduced food intake, resulting in fat loss in flies (Supplementary information, Fig. S1). Therefore, we used 5% sucrose medium as controls in the following studies when a simple food recipe was desirable, in particular for feeding-related assays.

Adding protein sources in food did not affect flies’ proboscis extension reflex (PER), the initial step of feeding behavior, suggesting that dietary proteins did not produce an aversive chemosensory signal (Supplementary information, Fig. S2a). The decrease in lipid storage upon HPD feeding would potentially reduce starvation tolerance. We thus examined behavioral differences of starved flies fed with HPD or 5% sucrose in prior. Using a previously developed video-based foraging paradigm,^33^ we found that starved flies previously fed with HPD reached food more quickly and stayed on food longer than those previously fed with 5% sucrose (Supplementary information, Fig. S2b–d). These HPD-fed flies also exhibited increased sucrose consumption upon starvation (Supplementary information, Fig. S2e). These results further confirmed that dietary protein intake significantly reduced flies’ energy storage and therefore their starvation tolerance.

### Cysteine was the key factor in HPD-induced body fat loss

**Fig. 2:**
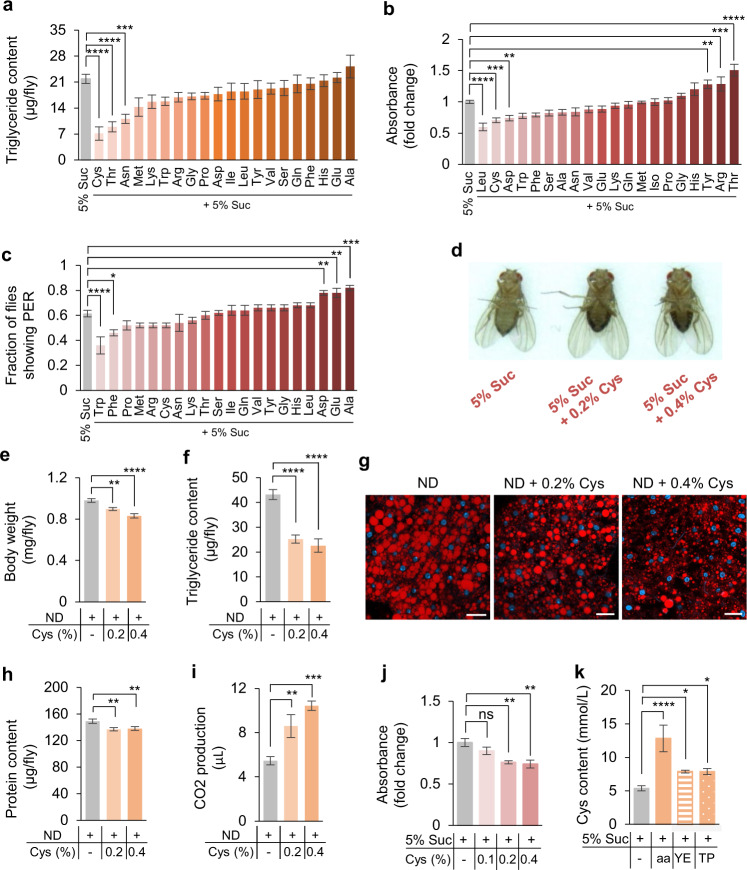
Dietary cysteine drove weight loss in a dose-dependent manner. **a** Triglyceride content of flies fed with 5% sucrose (grey) or 5% sucrose supplemented with 0.2% individual amino acid (orange) for 5 days (*n* = 5–8). **b** Food consumption of flies fed with 5% sucrose (grey) or 5% sucrose supplemented with 0.2% individual amino acid (red) for 5 days (*n* = 5–26). **c** Fraction of flies showing PER response to 5% sucrose supplemented with individual amino acid (*n* = 5–7 groups, 10 flies per group). **d**–**i** The metabolic effects of dietary cysteine feeding. Representative flies from each treatment group (**d**). Body weight (**e**, *n* = 7–9), triglyceride content (**f**, *n* = 8), Nile Red staining of the fat body (**g**, scale bars, 20 μm), protein content (**h**, *n* = 10), and 1 h CO_2_ production (**i**, *n* = 8–14). **j** Consumption of food containing dietary cysteine by pre-starved flies (*n* = 6–7). **k** Hemolymph cysteine levels from flies assayed in Supplementary information, Fig. S1a (*n* = 8–18). ns, *P* > 0.05; *P < 0.05; **P < 0.01; ***P < 0.001; ****P < 0.0001. One-way ANOVA followed by post hoc test with Bonferroni correction was used for multiple comparisons when applicable.

To determine which specific amino acid(s) in HPD might play a key role in reducing fat storage, we fed flies with 5% sucrose supplemented with individual amino acids for 5 days and assayed their lipid content. Amongst 20 natural L-amino acids we tested, only three of them, cysteine, threonine, and asparagine, showed effect in reducing body fat, whereas cysteine exhibited the most robust effect (Fig. 2a). We also tested the effect of these 20 amino acids on feeding behavior to identify the potential satiety signal in HPD. The result showed that addition of three amino acids including leucine, cysteine, and aspartate led to a significant decrease of food consumption (Fig. 2b). The inhibitory effect of cysteine on food consumption was specific to sugar, since protein consumption remained unchanged when cysteine was added (Supplementary information, Fig. S3a). The possibility that cysteine suppressed feeding as a direct aversive cue was excluded, as flies exhibited no change in PER to sugar or protein when cysteine was added (Fig. 2c; Supplementary information, Fig. S3b). We therefore chose to further investigate how dietary cysteine influenced fat storage and food intake.

We tested whether dietary cysteine drove fat loss in a dose-dependent manner. Flies were fed with 5% sucrose supplemented with different concentrations of cysteine for 5 days and their abdominal fat deposit decreased significantly (Fig. 2d). Furthermore, flies raised on ND plus dietary cysteine showed decreased body weight and fat storage, and both body weight (Fig. 2e) and fat storage (Fig. 2f, g) were inversely correlated with dietary cysteine concentrations. It is worth noting that body protein content remained stable upon cysteine feeding (Fig. 2h), suggesting that other amino acid(s) might modulate protein content (Fig. 1b). Similar to the effect of HPD feeding, body fat loss coincided with increased energy expenditure (Fig. 2i) and reduced food consumption (Fig. 2j) in a dose-dependent manner. These flies were behaviorally more sensitive to starvation, as evidenced by shorter latency to locate food (Supplementary information, Fig. S3c) and longer stay on food (Supplementary information, Fig. S3d, e), phenocopying the effect of HPD feeding (Supplementary information, Fig. S2b–d).

Therefore, we speculated that the fat loss effect of HPD was largely attributed to the cysteine content. We assayed hemolymph cysteine levels of flies fed with different types of HPD (Supplementary information, Fig. S1a). The results showed that all three HPD increased hemolymph cysteine levels, whereas amino acid mixture had the most significant effect (Fig. 2k). These results might explain the most robust fat loss effect of amino acid mixture compared to yeast extract and tryptone (Fig. 1c; Supplementary information, Fig. S1c).

### FMRFa signaling was required for cysteine-induced fat loss

**Fig. 3:**
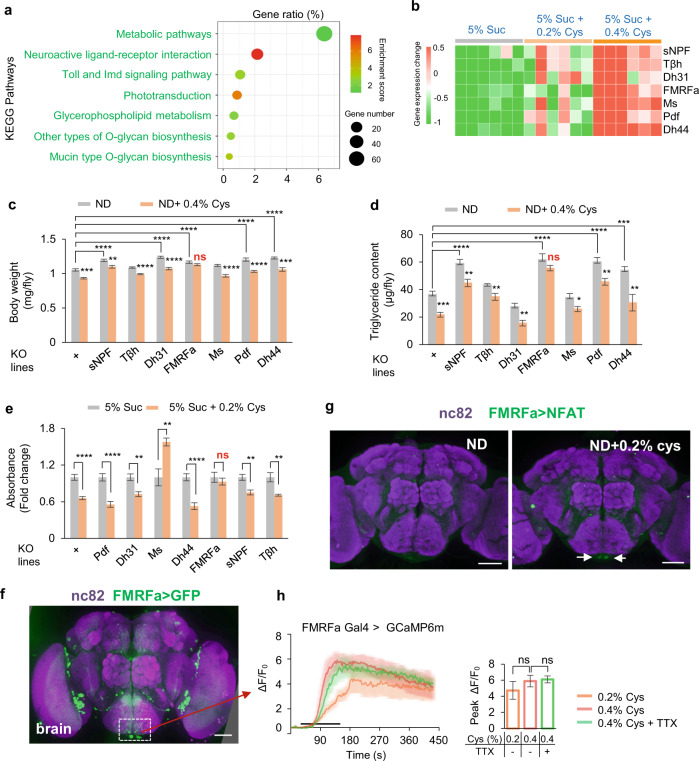
FMRFa signaling was required for lipid degradation upon dietary cysteine feeding. **a**, **b** RNA-seq analysis of flies fed with 5% sucrose or 5% sucrose supplemented with 0.2% cysteine for 5 days (*n* = 6). The top influenced signaling pathways by dietary cysteine from KEGG analysis (**a**). Heatmap of differentially expressed neuropeptide- and neurotransmitter-related genes (FDR < 0.001, fold change > 2). Red and green blocks represented upregulated and downregulated genes by cysteine feeding, respectively (**b**). **c**, **d** Body weight (**c**, *n* = 6–7) and triglyceride content (**d**, *n* = 6–8) of flies of the indicated genotypes fed with ND or ND supplemented with 0.4% cysteine for 5 days. **e** Food consumption of flies of the indicated genotypes fed with 5% sucrose or 5% sucrose supplemented with cysteine (*n* = 5–10). **f** FMRFa expression in the brain, as illustrated by mCD8::GFP expression driven by *FMRFa-GAL4*. Scale bar, 50 μm. **g** The activity of FMRFa^+^ neurons in the brain after cysteine feeding measured by the NFAT system. Scale bars, 50 μm. **h** Representative traces and quantification of ex vivo calcium responses of FMRFa^+^ neurons during the perfusion of cysteine with or without TTX (*n* = 6). ns, *P* > 0.05; *P < 0.05; **P < 0.01; ***P < 0.001; ****P < 0.0001. One-way and two-way ANOVA followed by post hoc test with Bonferroni correction were used for multiple comparisons when applicable.

It was reported that cysteine might modulate TOR signaling and flies’ metabolic activities.^46^ However, neither rapamycin feeding (an inhibitor of TOR) nor neuronal overexpression of dominant-negative TOR^TED^ affected cysteine-induced fat loss and feeding suppression (Supplementary information, Fig. S4). To investigate the mechanism underlying cysteine-induced fat loss, we carried out RNA-seq analysis of flies fed with dietary cysteine or not. Compared to sucrose-fed controls, ~1000 genes were significantly changed in cysteine-fed group (Supplementary information, Fig. S5a). By Kyoto Encyclopedia of Genes and Genomes (KEGG) analysis, we found that these differentially expressed genes were enriched in multiple metabolic and neurohormonal pathways (Fig. 3a).

Considering the importance of neurohormonal signaling in lipid metabolism,^47,48^ we focused on seven neuropeptide/neurotransmitter genes that were most influenced by dietary cysteine feeding (fold change > 2, FDR < 0.001, Fig. 3b). We collected knockout flies for these genes and assayed these flies with or without cysteine feeding. The results demonstrated that only *FMRFa*^*–/–*^ flies became unresponsive to dietary cysteine feeding and their body weight and fat storage remained unchanged (Fig. 3c, d). Besides, addition of cysteine in ND significantly enhanced FMRFa expression (Supplementary information, Fig. S5b). These results suggest that elevated FMRFa signaling was required for cysteine-induced fat loss. Interestingly, lipid droplets of these *FMRFa*^*–/–*^ flies were significantly larger than those of controls, suggesting that FMRFa was also involved in the regulation of lipid droplet biogenesis (Supplementary information, Fig. S5c).

HPD feeding also suppressed food consumption. We thus assayed the above knockout flies in our feeding assay and confirmed that only *FMRFa*^*–/–*^ flies showed diminished response to dietary cysteine (Fig. 3e). As a complementary approach, we also selected the receptor genes of those differentially expressed neuropeptides from our RNA-seq analysis (Fig. 3b) and tested them in a neuron-specific (*elav-GAL4*) RNAi screen. Among the lines we tested, only knocking down FMRFaR, the cognate receptor of FMRFa, eliminated the effect of dietary cysteine in suppressing food intake (Supplementary information, Fig. S6a, b). This result was further validated by using a different pan-neuronal GAL4 driver (*nsyb-GAL4*; Supplementary information, Fig. S6c). In line with the effect on feeding behavior, knocking down FMRFaR also suppressed cysteine-induced fat loss (Supplementary information, Fig. S6d). It was possible that neuronal FMRFa signaling had an indirect role in fat metabolism due to feeding suppression. Alternatively, FMRFa signaling within the nervous system might be critical for elevated FMRFa production upon cysteine feeding and hence its effect on fat metabolism.

We next sought to understand how dietary cysteine induced FMRFa signaling. By using *FMRFa-GAL4*, we found that FMRFa was expressed in the fly brain and the ventral nerve tube (VNC), but not in the gut, the fat body, or the proboscis (Fig. 3f; Supplementary information, Figs. S5d–f and S7a). To examine which group(s) of FRMFa^+^ neurons were activated by dietary cysteine, we used *FMRFa-GAL4* to express a transcriptional reporter system involving an NFAT-based tracing method (CaLexA) in which sustained neural activity drove GFP expression.^49^ In the brain, 2 neurons in the subesophageal zone (SEZ), a brain region housing the circuitry of gustatory sensory system and feeding behavior,^50^ showed enhanced GFP intensity upon cysteine feeding (Fig. 3g). We also performed calcium imaging in an ex vivo brain preparation^36^ and examined calcium responses in these two neurons upon the perfusion of cysteine. These neurons exhibited robust calcium transients upon cysteine stimulation (Fig. 3h). FMRFa^+^ neurons in the SEZ were likely directly activated by cysteine, because pre-treatment with tetrodotoxin (TTX), a blocker of voltage-gated Na^+^ channels that eliminated synaptic transmissions,^36^ had no effect on cysteine-induced calcium responses in these neurons (Fig. 3h). Collectively, these data suggest that FMRFa^+^ neurons in SEZ could directly sense dietary cysteine and impose suppressive effect on feeding behavior.

In the VNC, there were also a small number of FMRFa^+^ neurons that could be activated by cysteine, as demonstrated in the NFAT assay as well as calcium imaging (Supplementary information, Fig. S7b, c). Through extensive axon terminals, the neurons in the VNC exerted extensive control of peripheral tissues including the fat body.^51^ We thus speculated that these FMRFa^+^ cells in the VNC might modulate fat metabolism. Taken together, FMRFa signaling mediated the effect of dietary cysteine in reducing body fat storage and in suppressing food intake, possibly by directly sensing dietary cysteine and modulating fat metabolism and feeding circuitry, respectively.

### FMRFa enhanced lipolysis via PKA signaling in the fat body

**Fig. 4:**
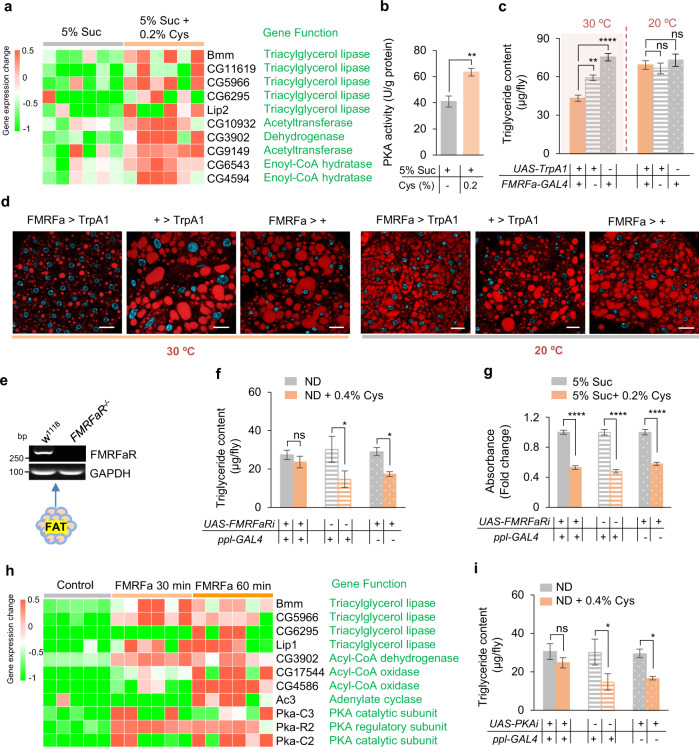
FMRFa activated PKA pathway. **a** RNA-seq analysis of the fat body of flies fed with 5% sucrose or 5% sucrose supplemented with 0.2% cysteine for 5 days (*n* = 6). A list of differentially expressed lipase genes was shown. **b** PKA enzymatic assay of the fat body tissue extract of flies fed with 5% sucrose or 5% sucrose supplemented with 0.2% cysteine for 5 days (*n* = 5). **c**, **d** Triglyceride content (**c**, *n* = 8–11) and Nile Red staining of the fat bodies (**d**) collected from flies of the indicated genotypes at different treatment temperatures (12 h at 30 °C; 20 °C). Lipids are in red and nucleus in blue. Scale bars, 20 μm. **e** RT-PCR analysis of FMRFaR expression in the fat bodies of *w*^*1118*^ and *FMRFaR*^*–/–*^ flies. **f** Triglyceride content of flies of the indicated genotypes fed with ND or ND supplemented with 0.4% cysteine for 5 days (*n* = 7–13). **g** Food consumption of flies fed with 5% sucrose (grey) or 5% sucrose supplemented with 0.2% cysteine (orange) for 5 days (*n* = 6). **h** RNA-seq analysis of the fat body of flies incubated with synthetic FMRFa (20 μg/mL) or not (*n* = 5–6). A list of differentially expressed lipase genes and PKA-related genes was shown. **i** Triglyceride content of flies of the indicated genotypes fed with ND or ND supplemented with 0.4% cysteine for 5 days (*n* = 6–13). ns, *P* > 0.05; *P < 0.05; **P < 0.01; ****P < 0.0001. Student’s *t*-test and one-way ANOVA followed by post hoc test with Bonferroni correction were used for multiple comparisons when applicable.

We next sought to examine the cellular signaling mechanism underlying dietary cysteine-induced metabolic and behavioral changes. We conducted RNA-seq of fat bodies from flies fed with or without cysteine and found that the differentially expressed genes were enriched in lipolysis pathways and other catabolic pathways (Supplementary information, Fig. S8a, b), indicating that dietary cysteine triggered catabolism in fat tissues. Among them, we observed that a large group of lipase genes were upregulated by cysteine feeding, including the rate-limiting lipase gene *brummer* (*bmm*, Fig. 4a). Consistently, the enzymatic activity of PKA, a known upstream regulator of *bmm*,^52^ was upregulated by dietary cysteine (Fig. 4b). Therefore, cysteine feeding might induce fat loss via promoting lipolysis in the fat body.

We then asked whether FMRFa signaling exerted a direct effect on lipolysis in the fat body. Activating FMRFa^+^ neurons by ectopic expression of a temperature-sensitive cation channel, *Drosophila* TRPA1, showed a significant decrease in fat mass and size of lipid droplets at 30 °C (Fig. 4c, d). We also examined the metabolic effect of synthetic FMRFa peptides. Upon abdominal injection of two different synthetic FMRFa peptides, CO_2_ production was significantly increased compared to flies injected with saline or an unrelated synthetic peptide, indicating an enhancement of catabolic metabolism by FMRFa signaling in the fat body (Supplementary information, Fig. S9a). Consistently, FMRFa injection also reduced body fat storage (Supplementary information, Fig. S9b, c).

As the direct downstream target of FMRFa, FMRFaR was shown to be expressed in the fat body of control but not *FMRFaR*^*–/–*^ flies (Fig. 4e). When FMRFaR was specifically knocked down in the fat body, dietary cysteine was unable to reduce fat storage (Fig. 4f), but still inhibited feeding behavior (Fig. 4g). These results suggest that FMRFaR in the fat body was responsible for the enhanced lipolysis and reduced fat storage, but not suppressed feeding, by dietary cysteine.

To further confirm a direct role of FMRFa signaling in lipolysis in the fat body, we isolated fat body tissues and stimulated the tissues with synthetic FMRFa peptide ex vivo, and subjected these tissue samples to RNA-seq analysis. The results showed that differentially expressed genes were enriched in the lipolysis and other catabolic pathways, similar to those induced by dietary cysteine (Fig. 4h; Supplementary information, Fig. S8c, d). These ex vivo experiments demonstrated a direct effect of FMRFa in promoting lipolysis in the fat body. Notably, FMRFa administration upregulated PKA pathways (Fig. 4h), consistent with the effect of dietary cysteine (Fig. 4b). We therefore tested whether blocking PKA signaling eliminated the fat loss effect of dietary cysteine. Indeed, when we specifically knocked down PKA in the fat body, cysteine feeding did not reduce body fat storage (Fig. 4i). Therefore, FMRFa signaling could directly induce lipolysis in the fat body via FMRFaR and PKA signaling.

### FMRFa signaling also mediated the behavioral effect of dietary cysteine

Besides its direct role in lipolysis, dietary cysteine also reduced food consumption, which might also contribute to the fat loss effect in an indirect manner. Given that FMRFa signaling through the fat body was not responsible for the behavioral effect of dietary cysteine (Fig. 4g), we sought to understand how FMRFa signaling mediated the behavioral effect of dietary cysteine.

**Fig. 5:**
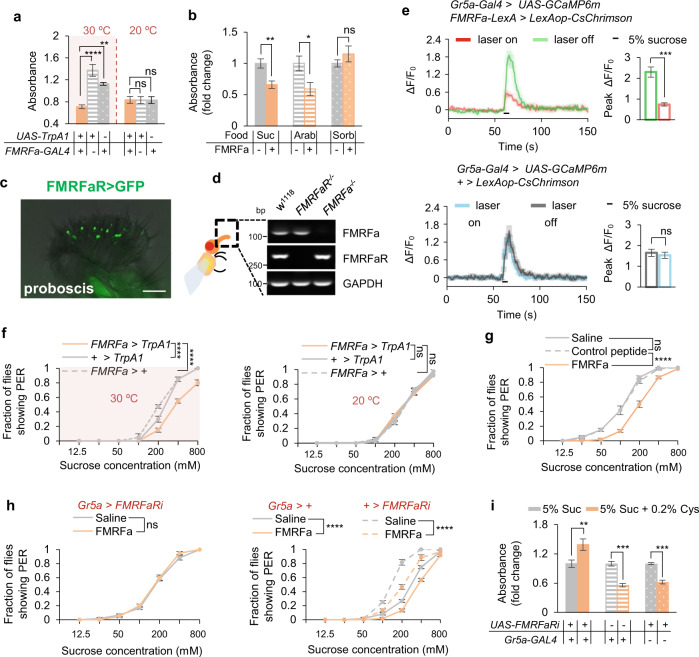
FMRFa suppressed feeding behavior by reducing sweet sensitivity. **a** Food consumption of flies of the indicated genotypes at different environmental temperatures (*n* = 5–6). **b** Food consumption of pre-starved flies assayed with the indicated sugar types. Flies were injected with saline or synthetic FMRFa 30 min before the assay (*n* = 6–8). **c** FMRFaR expression in the proboscis, as illustrated by mCD8::GFP expression driven by *FMRFaR-GAL4*. Scale bar, 50 μm. **d** RT-PCR analysis of the proboscis of flies for FMRFa and FMRFaR expression. **e** Representative traces (left) and quantification (right) of peak calcium transients of Gr5a^+^ neurons in the indicated flies upon feeding with 5% sucrose after the photo-activation of FMRFa^+^ neurons (*n* = 6–7). **f** Fraction of flies of the indicated genotypes at different environmental temperatures showing PER responses to different concentrations of sucrose (*n* = 4 groups, 10 flies per group). **g** Fraction of flies showing PER responses to different concentrations of sucrose (*n* = 6 groups, 10 flies per group). Flies were injected with saline or synthetic FMRFa 30 min before the assay. **h** Fraction of flies of the indicated genotypes showing PER responses to different concentrations of sucrose (*n* = 6 groups, 10 flies per group). Flies were injected with saline (grey) or synthetic FMRFa (orange) 30 min before the assay. **i** Food consumption of flies of the indicated genotypes (*n* = 5–8). ns, *P* > 0.05; *P < 0.05; **P < 0.01; ***P < 0.001; ****P < 0.0001. One-way and two-way ANOVA followed by post hoc test with Bonferroni correction were used for multiple comparisons when applicable.

When FMRFa^+^ neurons were artificially activated, flies exhibited decreased food consumption, phenocopying the effect of dietary cysteine (Fig. 5a). We then asked whether FMRFa ceased feeding via suppressing the appetitive perception of food, and/or via enhancing the internal satiety status. To distinguish between these two alternates, we fed flies with arabinose, a non-nutritive artificial sweetener, or sorbitol, a tasteless yet nutritive sugar. In the presence of dietary cysteine, arabinose consumption was reduced, whereas sorbitol consumption remained unchanged (Supplementary information, Fig. S10a), suggesting that dietary cysteine inhibited feeding behavior via suppressing the perception of sweet taste. In line with this explanation, flies injected with synthetic FMRFa exhibited reduction in sucrose and arabinose feeding, but not sorbitol feeding (Fig. 5b). Therefore, although dietary cysteine did not directly modulate appetitive responses (Fig. 2c), it could rather reduce sweet sensitivity via the induction of FMRFa signaling to gustatory neurons.

We thus asked whether sweet-sensing gustatory neurons could be directly modulated by FMRFa. FMRFaR showed expression in the proboscis, the VNC, the brain, and very limited expression in the gut and the fat body (Fig. 5c; Supplementary information, Fig. S10b–e). RT-PCR confirmed that FMRFaR was expressed in the proboscis, where Gr5a^+^ sweet-sensing gustatory neurons were located (Fig. 5d).^26^ Therefore, FMRFa^+^ neurons might exert its behavioral effect via FMRFaR expressed in Gr5a^+^ gustatory neurons.

Indeed, FMRFa^+^ neurons in the SEZ region of the fly brain exhibited local projections in the region (Fig. 3f, dotted line), where Gr5a^+^ neurons also project to.^53^ Furthermore, nSyb-GRASP analysis^54^ confirmed that FMRFa^+^ neurons and Gr5a^+^ neurons formed active synaptic innervations in the SEZ region (Supplementary information, Fig. S10f). Therefore, it is likely that these FMRFa^+^ neurons directly innervate and modulate Gr5a^+^ neurons in the SEZ region. Consistently, photo-activation of FMRFa^+^ neurons expressing CsChrimson^55^ significantly reduced sucrose-induced calcium responses in Gr5a^+^ neurons (Fig. 5e). Taken together, specific FMRFa^+^ neurons (e.g., those located in the SEZ (Fig. 3f, g)) were directly activated by dietary cysteine and suppressed the activity of Gr5a^+^ sweet-sensing gustatory neurons, which in turn suppressed food consumption.

In line with this hypothesis, we found that activating FMRFa^+^ neurons and FMRFa injection both led to decreased appetitive response in the PER assay (Fig. 5f, g). When FMRFaR expression was specifically knocked down in Gr5a^+^ neurons, FMRFa injection was unable to suppress PER towards sucrose (Fig. 5h), and dietary cysteine was unable to suppress food consumption (Fig. 5i). Taken together, these results suggest that besides its direct effect in promoting lipolysis in the fat body, FMRFa signaling also directly modulated sweet perception and food consumption via Gr5a^+^ gustatory neurons, both of which contributed to the fat loss effect of dietary cysteine.

### Dietary cysteine drove weight loss via NPFF, a mammalian RFamide peptide, in mice

**Fig. 6:**
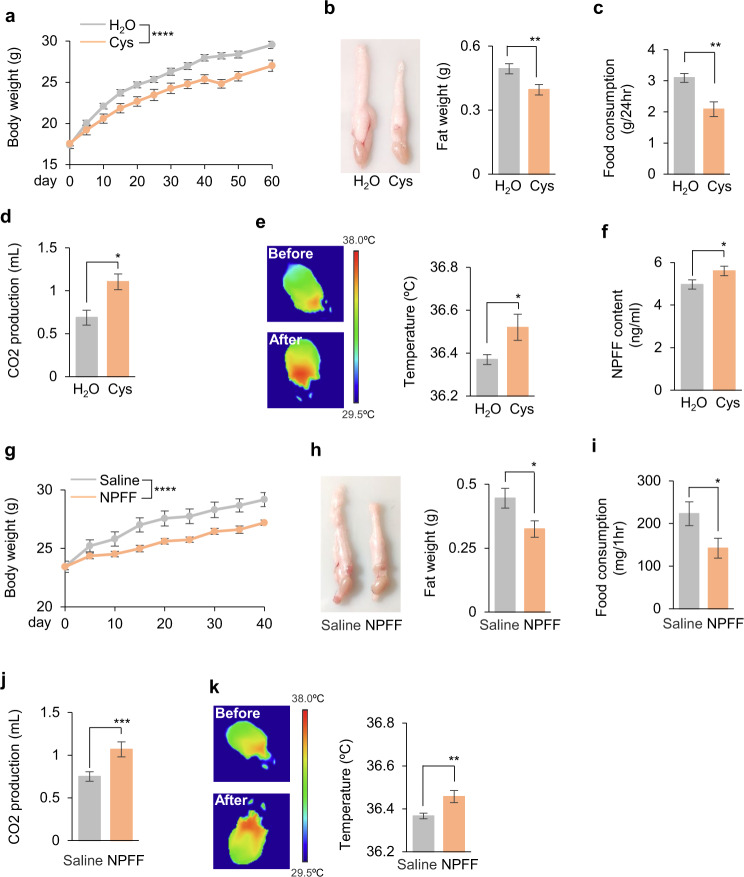
Dietary cysteine and NPFF drove weight loss in mice. **a**, **b** Body weight (**a**, *n* = 10) and epididymal fat pad (**b**, *n* = 10) of C57BL/6 mice orally gavaged with H_2_O alone or supplemented with cysteine (4 mmol/kg per day) for 60 days. **c**–**f** Food consumption (**c**, *n* = 10), CO_2_ production (**d**, *n* = 10), body temperature (**e**, *n* = 10), and blood NPFF levels (**f**, *n* = 20) of mice orally gavaged with H_2_O alone or supplemented with 4 mmol/kg of cysteine for 1 day. **g**, **h** Body weight (**g**, *n* = 10) and epididymal fat pad (**h**, *n* = 10) of mice with intravenous (IV) administration of NPFF (SQAFLFQPQRF-NH2, 3.2 mg/kg per 12 h) for 40 days. **i**–**k** Food consumption (**i**, *n* = 10), CO_2_ production (**j**, *n* = 10), and body temperature (**k**, *n* = 10) of mice with IV administration of NPFF (3.2 mg/kg once). ns, *P* > 0.05; *P < 0.05; **P < 0.01; ***P < 0.001; ****P < 0.0001. Student’s *t*-test and two-way ANOVA followed by post hoc test with Bonferroni correction were used for multiple comparisons when applicable.

Although HPD had been shown to reduce body weight in several species, it remained unknown whether they shared similar mechanisms as we discovered in flies in a cysteine- and FMRFa-dependent manner. To test this hypothesis, we gavaged mice with cysteine and observed a decrease in body weight and fat storage (Fig. 6a, b). Furthermore, dietary cysteine led to reduced food consumption (Fig. 6c) and upregulated energy expenditure as evidenced by the increase in CO_2_ production (Fig. 6d) and body temperature (Fig. 6e), recapitulating the effects that we observed in flies.

Among mammalian -RFamide peptides, NPFF was found to be involved in regulation of feeding behavior.^56^ Despite the lack of overall sequence similarity, the signature amino acid sequence -Arg-Phe-NH2 (-RFamide) was conserved between mouse NPFF and fly FMRFa^31,57^ (Supplementary information, Fig. S11a). We thus hypothesized that they might exert similar physiological functions. Supporting this claim, dietary cysteine enhanced NPFF production in mice (Fig. 6f), and NPFF injection caused fat loss and feeding suppression in flies, similar to the effect of FMRFa injection in flies (Supplementary information, Fig. S11b, c). We then asked whether NPFF injection could cause similar effects in mice. Indeed, NPFF injection reduced body weight and fat storage in mice (Fig. 6g, h). Furthermore, NPFF injection led to reduced food consumption (Fig. 6i) and upregulated energy expenditure (Fig. 6j, k).

Similar to flies, mice fed with dietary cysteine or injected with NPFF exhibited robust food-seeking behavior when subjected to starvation, again confirming the reduced energy storage and enhanced energy expenditure (Supplementary information, Fig. S11d–j). Thus, dietary cysteine exerted robust fat loss effect in mice via suppressing food consumption and promoting energy expenditure, which might be attributed to NPFF.

### Dietary cysteine and FMRFa/NPFF signaling improved insulin sensitivity

**Fig. 7:**
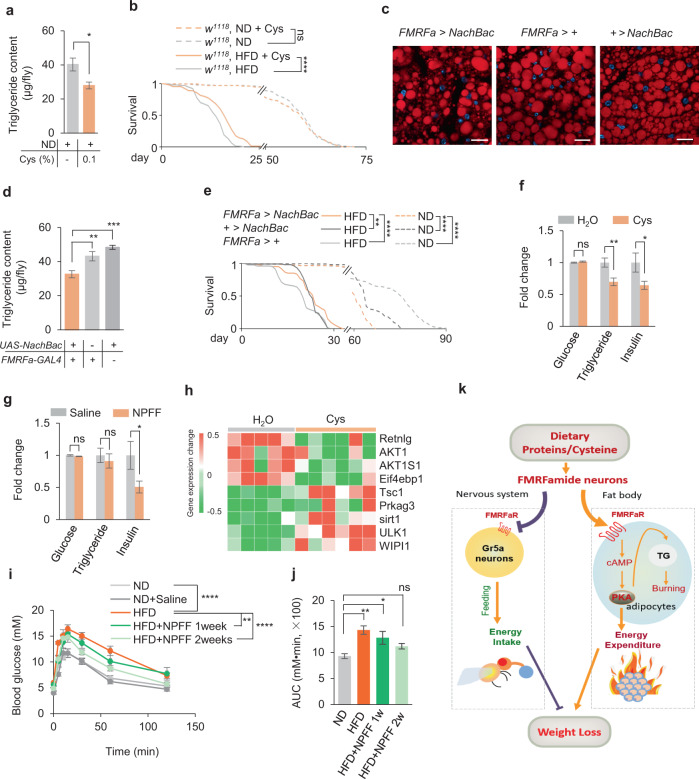
Dietary cysteine and FMRFa/NPFF provided partial protection against metabolic stress. **a**, **b** Triglyceride content (**a**, *n* = 8–9) and survival curve (**b**, *n* = 127–130) of flies fed with ND or HFD in the presence or absence of 0.1% cysteine. **c** Nile Red staining of the fat bodies. Scale bars, 20 μm. **d** Triglyceride content of flies of the indicated genotypes (*n* = 8). **e** Survival curve of flies of the indicated genotypes fed with ND or HFD (*n* = 101–121). **f** Blood glucose, triglyceride, and insulin levels of C57BL/6 mice orally gavaged with H_2_O or cysteine (4 mmol/kg per day) for 40 days (*n* = 10). **g** Blood glucose, triglyceride, and insulin levels of C57BL/6 mice with IV administration of NPFF (3.2 mg/kg per 12 h) for 40 days (*n* = 10). **h** RNA-seq analysis of the adipose tissue of mice orally gavaged with H_2_O or cysteine (*n* = 5–6). A list of differentially expressed insulin signaling-related genes was shown. **i**, **j** Oral glucose tolerance assay of mice fed with ND or HFD with IV administration of NPFF (*n* = 5). The Area Under the Curve (AUC) was shown in **j**. **k** A working model. Upon dietary protein intake (cysteine being the key ingredient), the secretion of neuropeptide FMRFa is increased. FMRFa exerts dual effects via its cognate receptor FMRFaR in two different organs: in the nervous system it reduces the sweet sensitivity of Gr5a^+^ gustatory neurons, leading to a suppression of energy intake; in the fat body, it triggers lipid degradation and increases energy expenditure via activating PKA signaling pathway. Overall, dietary cysteine intake represents a special satiety state in flies and mice and leads to body fat loss. ns, *P* > 0.05; *P < 0.05; **P < 0.01; ***P < 0.001; ****P < 0.0001. Student’s *t*-test, one-way and two-way ANOVA followed by post hoc test with Bonferroni correction were used for multiple comparisons when applicable.

Besides fat loss, we wondered whether dietary cysteine could improve systematic metabolic dysfunctions commonly seen in obese and overweight animals. We used a reduced cysteine concentration (0.1%) in long-term feeding experiments in flies to reduce potential side effect, and found that such diet induced a reduction in fat storage without affecting flies’ overall survival (Fig. 7a, b). Upon HFD feeding, flies’ survival was significantly reduced, which could be partially rescued by the addition of dietary cysteine (Fig. 7b). Chronic activation of FMRFa^+^ neurons by NaChBac, a bacterial sodium channel, reduced the fat content similar to cysteine feeding (Fig. 7c, d). Under ND conditions, chronic activation of FMRFa^+^ neurons led to reduced survival, likely due to insufficient food intake and fat storage (Fig. 7e, dotted lines). However, under HFD conditions, chronic activation of FMRFa^+^ neurons offered subtle but significant increase in lifespan (Fig. 7e, solid lines). Thus, low dose of dietary cysteine and enhanced FMRFa signaling conferred survival benefits under over-nutrition conditions like HFD. One common side effect of current weight-loss regimen is mental disorders such as anxiety and sleep loss. We found that dietary cysteine and FMRFa signaling did not alter flies’ overall locomotor activity or sleep duration/frequency (Supplementary information, Fig. S12). These results suggest that dietary cysteine and FMRFa treatment could be a safe way to reduce body fat without potential harm to lifespan or behavioral control.

We also examined the potential metabolic effect of cysteine/NPFF in mice. Upon cysteine feeding, fat storage and circulating insulin level were significantly reduced while blood sugar level remained stable (Fig. 7f). NPFF injection showed similar effects to cysteine feeding (Fig. 7g). Thus, both cysteine and NPFF might improve insulin sensitivity. Mechanistically, RNA-seq analysis of the adipose tissue revealed that differentially expressed genes were indeed enriched in insulin-related pathways. Among them, downregulated AKT and 4EBP1 and upregulated TSC1 upon cysteine feeding both indicated that mTOR pathway was suppressed (Fig. 7h). Evidently, genes in the autophagy pathway, a known suppressive regulator of mTOR signaling, were upregulated (*ULK1* and *WIPI1*, Fig. 7h). Meanwhile, the activation of AMPK pathway, as evidenced by the elevated *Prkag3* and *sirt1* (Fig. 7h), and the decreased expression of *Retnlg*, an insulin resistance-promoting factor, might also contribute to the improved insulin sensitivity in mice upon cysteine feeding.

Insulin resistance was tightly associated with metabolic disorders, particularly type 2 diabetes and cardiovascular diseases. We wondered whether NPFF protected HFD-fed mice from insulin resistance. Indeed, short-term injection of NPFF remarkably improved glucose tolerance in HFD-fed mice (Fig. 7i, j), indicating a protective role of NPFF against diet-induced metabolic diseases.

## Discussion

Obesity is developing into a prevailing and devastating health problem worldwide. Despite the development of several surgical and pharmaceutical interventions, we need a safe, low-cost, and easy-to-use way to help the public control their body weight and reduce the risk of developing metabolic diseases. Lifestyle interventions, such as diet restriction, ketogenic diet, and HPD, are of potential value. However, the side effects greatly limit their practical application. Diet restriction and ketogenic diet cause consumption of body protein and fat simultaneously, and consequently significant muscle loss.^58^ Ketogenic diet and HPD also cause kidney and other organ diseases due to the accumulation of harmful metabolites. In the present study we first confirmed that three types of HPD all exhibited robust fat loss effect in fruit flies via suppressing food intake and promoting lipid degradation. We then screened 20 individual natural amino acids and identified cysteine as the only amino acid that recapitulated the effects of HPD in a dose-dependent manner (Fig. 7k). Dietary cysteine seemed to be safe with no obvious effect on flies’ overall metabolism, behavior, and survival. These results might help develop safe and effective diet supplement for weight management.

In fruit flies, dietary cysteine promoted the production of FMRFa, a neuropeptide terminated by the signature sequence Phe-Met-Arg-Phe-NH2. FMRFa signaling has been shown to regulate flight,^59^ escape response,^60^ stress-induced sleep,^61^ and muscle contraction.^62^ *FMRFa* knockout and RNAi-mediated knockdown of FMRFaR both blocked the fat loss effect of dietary cysteine. FMRFaR, the cognate receptor of FMRFa, mediated both the feeding suppression and the fat degradation effects of dietary cysteine. FMRFa directly triggered fat degradation in the fat body via FMRFaR in a PKA-dependent manner, and also directly reduced sweet sensitivity in Gr5a^+^ gustatory neurons and hence food consumption (Fig. 7k). Similar to dietary cysteine, manipulating FMRFa-FMRFaR signaling significantly improved survival upon HFD feeding, but did not affect flies’ locomotion and sleep. Therefore, besides dietary cysteine supplement, other novel fat loss interventions might also be developed to target FMRFa-FMRFaR signaling.

NPFF is a mammalian RFamide peptide. NPFF is involved in the regulation of various physiological functions in vivo, including pain sensation, body temperature control, cardiovascular function, gastrointestinal movement, and feeding behavior.^63^ We therefore examined whether and how dietary cysteine and NPFF signaling exhibited similar effects in mice. In mice, cysteine feeding promotes NPFF expression, which also reduces body weight by inhibiting feeding and promoting fat degradation. Neither cysteine feeding nor NPFF injection affected circulating glucose levels, suggesting low risk of hypoglycemia. Meanwhile, the decrease of insulin level suggested that both manipulations improved insulin sensitivity, which was confirmed by glucose tolerance assays. These results further validated the therapeutic potential of dietary cysteine supplement and NPFF manipulations.

Notably, two NPFF receptors NPFFR1 (G protein-coupled receptor 147, GPR147) and NPFFR2 (GPR74) are present in human genome.^64^ There is an emerging interest in the lipid-lowering effect of GPR family proteins. Recent studies have found that cold stimulation can upregulate GPR3, and the polymerization of GPR3 can activate the intracellular cAMP pathway to degrade fat.^65^ Another study has shown that a loss-of-function mutation in the *GPR75* gene leads to > 50% lower probability of obesity in human, indicating that inhibiting GPR75 could be a potential weight loss approach.^66^ GPR146 plays a similar role in lipid metabolism to GPR75, and GPR146 knockout effectively reduces plasma cholesterol levels in mice.^66^ Our findings suggested that the two NPFF receptors, GPR147/GPR74, might be a new target for weight loss.

Besides the therapeutic potential, our results also identified FMRFa as a novel satiety signal triggered by dietary cysteine. Conventionally, the satiety state usually arises after sufficient food consumption and adequate nutrient storage at the organismal level. Circulating energy cues (such as glucose and amino acids), gastrointestinal signals (such as CCK, GLP-1), and adiposity signals (such as leptin and insulin) are all well documented satiety signals, which can be integrated in the nervous system (e.g., in the mammalian hypothalamus) and effectively modulate energy intake and expenditure. Many of these satiety signals are conserved in many animal species such as human, rodents, and flies.^25,28,67–74^ Unlike these signals, dietary cysteine intake and FMRFa/NPFF signaling represent a special and “artificial” satiety state. Without excess food consumption or nutrient storage, dietary cysteine and FMRFa/NPFF signaling induce suppression of food consumption and enhancement of energy expenditure, and hence fat loss effect. For future studies it is of great value to investigate how elevated cysteine level is sensed and processed in the nervous system and how it interacts with other satiety pathways.

## Materials and methods

### Animals

Flies were kept on standard fly food at 60% humidity and 25 °C on a 12-h light/12-h dark cycle. Virgin female flies were selected shortly after eclosion and placed in standard fly food (ND) vials (20 flies per vial) for 5–6 days till experiments. HFD-fed flies were raised in vials with HFD medium containing 80% ND and 20% coconut oil. For experiments that required dTRPA1, flies were grown at 18 °C for 7–8 days before being transferred to 30 °C to start assay.

The following UAS-RNAi lines: *UAS-sNPF-R RNAi* (#27507), *UAS-Pdf-R RNAi* (#38347), *UAS-Dh31-R RNAi* (#25925), *UAS-FMRFaR RNAi* (#25858), *UAS-Ms-R1 RNAi* (#27529), *UAS-Ms-R2 RNAi* (#25832), *UAS-Dh44-R1 RNAi* (#28780), *UAS-Tβh RNAi* (#27667), *LexAop-nSyb-spGFP1-10,UAS-CD4-spGFP11* (#64315), *UAS-GCaMP6m* (#42748), *Gr5a-GAL4* (#57592), *ppl-GAL4* (#58768), *elav-GAL4* (#25750), and *FMRFa-GAL4* (#56837) were obtained from the Bloomington *Drosophila* Stock Center at Indiana University. *FMRFa-LexA*, *FMRFaR-GAL4*, and all knockout lines were from Yi Rao^75^ (Capital Medical University, Beijing). *UAS-dTRPA1* and *nsyb-GAL4* were from David Anderson (Caltech). *UAS-GCaMP6m,LexAop-CsChrimson* was from Yufeng Pan (Southeast University, Nanjing)*. UAS-PKA* *RNAi* (#5749) was from the Tsinghua Fly Center.

Four-week-old C57BL/6J male mice were obtained from the Experimental Animal Center of Chongqing Medical University. All mice were housed under standard laboratory conditions at 22 °C in a 12-h light/12-h dark cycle. All procedures were performed in accordance with national and international guidelines, established by Laboratory Animal Welfare and Ethics Committee of Chongqing University (No. CQU-IACUC-RE-202108-001).

### Behavioral assays (fly)

For feeding assay,^38^ 5-day-old flies were starved on 2% agar for 24 h, and then transferred to new vial with test food containing 0.5% Brilliant Blue (Sangon Biotech, China) for 10 min. After a quick freeze (–20 °C), flies were decapitated in PBS, and then were homogenized and centrifuged (13,000× *g*, 5 min). The supernatants were diluted with PBS to a total of 1 mL. The absorbance was measured at 620 nm.

PER was assayed as described previously.^76^ Briefly, individual flies were gently aspirated and introduced into a 200 μL pipette tip. Flies were first sated with water and then subjected to different sucrose solutions with each solution tested twice. Flies showing PER response to at least one of the two trials were considered positive to the concentration.

DAMS-based locomotion and sleep assays were performed as described in our earlier work.^77^ Briefly, individual flies were lightly anesthetized and then introduced into 5 mm × 65 mm polycarbonate tubes (Trikinetics). One end of these tubes was filled with food containing 5% (wt/vol) sucrose + 2% (wt/vol) agar and the other end was blocked using cotton wool. These tubes were then inserted into DAMS monitors and placed in fly incubators. The frequency of these flies through the middle of the tube was counted by an infrared beam as an indirect measure of their locomotion. Sleep was defined as a period of inactivity lasting at least 5 min.

The food seeking assay based on video recording was performed as described previously.^33^ Briefly, individual flies were starved for the indicated time, and transferred into a behavioral chamber (84 mm × 84 mm) with food (5% sucrose) in the center. Fly locations were recorded by a camera on top of the chamber with a 640 × 480 resolution every 1 s and further calculated by a custom computer program based on Pysolo to analyze the positions and moving trajectories of the fly during the assay. The time that the fly first located on food area for > 3 s was defined as foraging time.

### Behavioral assays (mouse)

Food-seeking model was performed as Supplementary information, Fig. S11d. The open area consisted of custom chamber (50 cm × 30 cm) enclosure. Foods in a cup were placed in a corner of the open area, which were surrounded with electric grids by means of which a 0.25 mA shock could be delivered to the mouse. Before experiments, mice were trained in this chamber to find the food region and electric grids, in a condition without electric grids in the first day and with electric grids in the next day. After training, individual mouse was pre-starved for 12 h, and then placed in the chamber. The mouse’s position-tracking data were recorded by a camera every 1 s and calculated by a custom computer program. The time that the mouse first located on food area for > 3 s was defined as foraging time.

### Biochemical assays

The following kits were used in this study: glucose (BC2490, Solarbio Life Sciences, China), triglyceride (for blood lipid detection, A110-1-1, Nanjing Jiancheng Bioengineering Institute, China), triglyceride (for fly detection, D799795-0050, Sangon Biotech, China), cysteine (E-BC-K352-M, Elabscience, China) and insulin (SEKM-0141, Solarbio Life Sciences, China). All the manipulations were according to the manufacturer’s instructions.

### Oral glucose tolerance tests

Oral glucose tolerance tests were performed on male mice that had been fasted for 12 h. 10% glucose (0.2 mL per mouse) was delivered into the stomach, and glucose levels were determined in blood harvested from the tail tip using Accu-Chek blood glucose monitoring systems (Roche, Switzerland), immediately 0 min, 5 min, 10 min, 15 min, 30 min, 60 min and 120 min after the injection.

### Hemolymph extraction and cysteine measurement

Forty flies were decapitated and transferred to a 0.5 mL punctured tube. The tube was then placed into a new 1.5 mL Eppendorf tube and subjected to centrifugation at 2500× *g* at 4 °C for 10 min. Hemolymph was collected in 1.5 mL tube and used for cysteine measurement immediately. 4 μL hemolymph per sample was analyzed for cysteine measurement. All the manipulations were according to the manufacturer’s instructions (E-BC-K352-M, Elabscience, China).

### PKA measurement

Fat bodies of flies were dissected in PBS with 10% phosphatase inhibitor complex II (Sangon, China). The abdominal carcass was technically challenging to be completely removed from fat bodies, but we tried to carefully remove the carcass as much as possible. The tissues were disrupted by Tissuelyser-32 (Jingxin, China). After centrifugation for 5 min at 5000× *g* at 4 °C, the supernatant was measured by the Insect PKA ELISA Kit (ml062773, Mlbio, China).

### Measurement of CO_2_ production

For flies, as described previously,^78^ 1 mL plastic tips were used for the respirometers with a small amount of CO_2_ absorbent (Soda lime, Sigma) between two pieces of sponge in the body of the tip. A 5 μL fine graduated capillary was connected to the plastic adaptor attached to the tip. Five flies were gently aspirated into the tip body. Then the tip was sealed to form a closed chamber. After being kept on the flat surface at 25 °C for 15 min to equilibrate, a little ink was injected into the end of the micropipet. The CO_2_ production was calculated based on the rate of movement of the ink.

For mouse, individual animal was kept in a closed chamber, which was pre-filled with 3 g Soda lime. Then the closed chamber was connected with water using a hose. After 10 min, the CO_2_ production was calculated based on the rate of movement of the water in the hose.

### Microinjection

Flies were first transferred and immobilized in a 200 μL pipette tip with their heads towards the end of the tip. Then the tip was cut open to expose the heads and a part of the thorax. ~20 nL of sterile saline with or without the addition of synthesized FMRFa-1# (GRDPKQDFMRF-NH2, 1 mg/mL), FMRFa-2# (DPKQDFMRF-NH2, 1 mg/mL), NPFF (SQAFLFQPQRF-NH2, 1 mg/mL) and control peptide (DYKDDDDKYPYDVPDYA, 1 mg/mL) was injected into the thorax of these flies with a glass micropipette using a microinjector (3-000-207, Drummond Scientific Company Instruments). The glass micropipette was pulled from thick-walled borosilicate capillaries (3-000-203-G/X, Drummond Scientific Company Instruments).

### Survival assays

Survival analysis was performed using female flies (*n* > 100) kept in independent vials. Ten flies per vial were transferred to fresh food every day. Each genotype or treatment was implemented twice. Survival curves were analyzed by the GraphPad Prism software (La Jolla, CA, USA). Statistical differences of survival curves were analyzed by log-rank test.

### Lipid droplet staining

Fat bodies of anesthetized flies were dissected in PBS and fixed in 4% formaldehyde at room temperature for 30 min. After washing thrice in PBS, the tissues were stained with Nile Red (2 ng/μL, Sangon, China) for 1 h and DAPI (20 μg/μL, Sangon, China) for 5 min. Images were captured on a confocal microscope (Carl Zeiss).

### Immunofluorescence staining

Fly samples were dissected in PBS on ice and transferred to 4% formaldehyde for fixation for 60 min. Fixed tissues were permeabilized and blocked with Dilution/Blocking Buffer (PBS containing 10% Calf Serum and 0.5% Triton X-100) for 2 h at room temperature. After blocking, the tissues were incubated with appropriate primary antibodies in Dilution/Blocking Buffer for 24 h at 4 °C. Then, these samples were washed 4 times with Washing Buffer (0.5% Triton X-100 in PBS) for 60 min at room temperature and subsequently incubated with secondary antibodies for 24 h at 4 °C. The samples were washed with Washing Buffer four times before being mounted in Fluoroshield (Sigma-Aldrich).

Images were acquired on a scanning confocal microscope with Olympus 20×/0.7 and 40×/0.95w. Antibodies were used at the following dilutions: mouse anti-nc82 (1:100, DSHB), rabbit anti-GFP (1:100, Abcam), Alexa Fluor 647 goat anti mouse (1:100, Cell Signaling Technology), Alexa Fluor 488 goat anti rabbit (1:100, Cell Signaling Technology).

### nSyb-GRASP

For detection of nSyb-GRASP signals, the fly brains were dissected in PBS and fixed in 4% formaldehyde for 60 min at room temperature. After fixation, the samples were washed, mounted and dehydrated as described in “Immunofluorescence staining”. Images were acquired using Olympus 20×/0.7 and 40×/0.95w.

### Calcium imaging

Fly brains or VNC was freshly dissected into the AHL buffer (108 mM NaCl, 8.2 mM MgCl_2_, 4 mM NaHCO_3_, 1 mM NaH_2_PO_4_, 5 mM KCl, 2 mM CaCl_2_, 80 mM sucrose, 5 mM HEPES, pH 7.3). The samples in the AHL buffer were recorded in 0.5 min to generate a base line. Then the solutions were changed to AHL + cysteine with the pH adjusted back to 7.3 by gentle perfusion for 2 min. Solutions in the perfusion chamber were controlled by a valve commander (Scientific Instruments). After stimulation, samples were washed out again with AHL. For the TTX test, 2 μM TTX was added to the AHL solution. Before the assay, the samples ware pre-treated with 2 μM TTX for 15 min.

All imaging studies were performed on an Olympus confocal microscope (FVMPE-RS) with a water immersion objective lens (25×/1.05w MP). Image analyses were performed in ImageJ and plotted in Excel (Microsoft). The ratio changes were calculated using the following formula: ΔF/F = (F – F0)/F0, where F is the mean florescence of cell body, F0 is the average base line (before stimulation).

### Optogenetics and in vivo calcium imaging

Newly enclosed virgin female flies were collected in a fresh vial with standard medium for 3 days and then transferred into a vial with regular food containing 400 μM all-trans-retinal (Sigma, R2500) for 2–4 days till experiments. These flies were anesthetized on ice and then glued onto a transparent tape. The cuticle of the dorsal part of the fly head was gently removed with forceps and the exposed brain was bathed in AHL. To activate the neurons expressing CsChrimson, the red LED (M625L3, Thorlabs) was placed above the brain, which was controlled by a custom computer program during opto-activation (0.5 s on and 0.5 s off for 3 min). After opto-activation, liquid food was delivered to the proboscis of flies by a micromanipulator (MP225, Sutter Instruments). The calcium signals were recorded at 1 frame/s.

### RNA-seq and data analysis

For RNA-seq of fly tissue or mouse tissue, total RNA was extracted using the Trizol reagent (Invitrogen, USA), subjected to poly(A) mRNA selection, cDNA synthesis, library preparation according to the manufacturer’s instructions (Vazyme, NR605), and sequencing (Illumina HiSeq X Ten platform).

For analysis, sequence data were subsequently mapped to *Drosophila*/mouse genome and uniquely mapped reads were collected. Transcript abundances were calculated by the FPKM (Fragments Per Kilobase of exon per Million fragments mapped). Transcripts with fold change > 2 and *P*-value < 0.05 were considered as differentially expressed. Functional annotation of differentially expressed genes was assessed by KEGG (http://www.genome.jp/kegg) and Gene Ontology (GO) (http://www.geneontology.org).

### Quantitative RT-PCR

Total RNA was isolated from fly tissue (for proboscis, 50 proboscises were prepared for one sample) with TRIzol reagent (Invitrogen, USA). The purified RNA products were then reverse transcribed with TransScript cDNA Synthesis SuperMix (TransGen). The quantitative PCR experiments were performed with SYBR premix Ex TaqTM (Takara, China) on a CFX96 Real-Time System (BioRad). The primers used are as follows: FMRFaR F: 5′-TTTCCGCAGAACAACAGT-3′, FMRFaR R: 5′-TTCCAAACAGCAGGATAGA-3′; FMRFa F: 5′-GTGCGCTCCGGGAAGATGGA-3′, FMRFa R: 5′-ACTGCGTTCGCCTGGGTTGC-3′; GAPDH F: 5′-GAATCCTGGGCTACACCG-3′, GAPDH R: 5′-CTTATCGTTCAGCGAAATGC-3′.

### Statistical analysis

Data presented in this study were verified for normal distribution by D’Agostino–Pearson omnibus test. Student’s *t*-test, one-way ANOVA and two-way ANOVA (for comparisons among three or more groups and comparisons with more than one variant) were used. The post hoc test with Bonferroni correction was performed for multiple comparisons following ANOVA.

## Supplementary information

Supplementary information, Fig. S1

Supplementary information, Fig. S2

Supplementary information, Fig. S3

Supplementary information, Fig. S4

Supplementary information, Fig. S5

Supplementary information, Fig. S6

Supplementary information, Fig. S7

Supplementary information, Fig. S8

Supplementary information, Fig. S9

Supplementary information, Fig. S10

Supplementary information, Fig. S11

Supplementary information, Fig. S12

## References

[1] Panuganti, K. K., Nguyen, M. & Kshirsagar, R. K. Obesity. in *StatPearls*. Treasure Island (FL): StatPearls Publishing (2022).

[2] Obesity and overweight. https://www.who.int/news-room/fact-sheets/detail/obesity-and-overweight (2021).

[3] Sanyal A (2017). Interplay between obesity-induced inflammation and cGMP signaling in white adipose tissue. Cell Rep.

[4] Shoelson SE, Lee J, Goldfine AB (2006). Inflammation and insulin resistance. J. Clin. Invest..

[5] Lopresti AL, Drummond PD (2013). Obesity and psychiatric disorders: commonalities in dysregulated biological pathways and their implications for treatment. Prog. Neuropsychopharmacol. Biol. Psychiatry.

[6] Rueda-Clausen CF, Ogunleye AA, Sharma AM (2015). Health benefits of long-term weight-loss maintenance. Annu. Rev. Nutr..

[7] Magkos F (2016). Effects of moderate and subsequent progressive weight loss on metabolic function and adipose tissue biology in humans with obesity. Cell Metab..

[8] Farhana, A. & Rehman, A. Metabolic consequences of weight reduction. in *StatPearls*. Treasure Island (FL): StatPearls Publishing (2022). 34283511

[9] Colquitt JL, Pickett K, Loveman E, Frampton GK (2014). Surgery for weight loss in adults. Cochrane Database Syst. Rev..

[10] Rebello CJ, Greenway FL (2020). Obesity medications in development. Expert Opin. Investig. Drugs.

[11] Kanoski SE, Hayes MR, Skibicka KP (2016). GLP-1 and weight loss: unraveling the diverse neural circuitry. Am. J. Physiol. Regul. Integr. Comp. Physiol..

[12] Kharitonenkov A (2005). FGF-21 as a novel metabolic regulator. J. Clin. Invest..

[13] Onakpoya IJ, Heneghan CJ, Aronson JK (2016). Post-marketing withdrawal of anti-obesity medicinal products because of adverse drug reactions: a systematic review. BMC Med..

[14] Mahu I (2020). Brain-sparing sympathofacilitators mitigate obesity without adverse cardiovascular effects. Cell Metab..

[15] Franz MJ (2007). Weight-loss outcomes: a systematic review and meta-analysis of weight-loss clinical trials with a minimum 1-year follow-up. J. Am. Diet. Assoc..

[16] Manore MM (2015). Weight management for athletes and active individuals: a brief review. Sports Med..

[17] Moon J, Koh G (2020). Clinical evidence and mechanisms of high-protein diet-induced weight loss. J. Obes. Metab. Syndr..

[18] Belza A (2013). Contribution of gastroenteropancreatic appetite hormones to protein-induced satiety. Am. J. Clin. Nutr..

[19] Wren AM (2001). Ghrelin enhances appetite and increases food intake in humans. J. Clin. Endocrinol. Metab..

[20] Cuenca-Sanchez M, Navas-Carrillo D, Orenes-Pinero E (2015). Controversies surrounding high-protein diet intake: satiating effect and kidney and bone health. Adv. Nutr..

[21] Nassel DR, Wu SF (2022). Cholecystokinin/sulfakinin peptide signaling: conserved roles at the intersection between feeding, mating and aggression. Cell Mol. Life Sci..

[22] Nassel DR, Zandawala M (2020). Hormonal axes in *Drosophila*: regulation of hormone release and multiplicity of actions. Cell Tissue Res..

[23] Kim SK, Rulifson EJ (2004). Conserved mechanisms of glucose sensing and regulation by *Drosophila* corpora cardiaca cells. Nature.

[24] Chatterjee, N. & Perrimon, N. What fuels the fly: Energy metabolism in *Drosophila* and its application to the study of obesity and diabetes. *Sci. Adv.* **7***,* eabg4336, (2021). 10.1126/sciadv.abg4336 PMC8189582 34108216

[25] Root CM, Ko KI, Jafari A, Wang JW (2011). Presynaptic facilitation by neuropeptide signaling mediates odor-driven food search. Cell.

[26] Dahanukar A, Lei YT, Kwon JY, Carlson JR (2007). Two Gr genes underlie sugar reception in *Drosophila*. Neuron.

[27] Inagaki HK, Panse KM, Anderson DJ (2014). Independent, reciprocal neuromodulatory control of sweet and bitter taste sensitivity during starvation in *Drosophila*. Neuron.

[28] Lin S, Senapati B, Tsao CH (2019). Neural basis of hunger-driven behaviour in *Drosophila*. Open Biol..

[29] Reiter LT, Potocki L, Chien S, Gribskov M, Bier E (2001). A systematic analysis of human disease-associated gene sequences in *Drosophila melanogaster*. Genome Res..

[30] Musselman LP (2011). A high-sugar diet produces obesity and insulin resistance in wild-type *Drosophila*. Dis. Model Mech..

[31] Nassel DR, Zandawala M (2019). Recent advances in neuropeptide signaling in *Drosophila*, from genes to physiology and behavior. Prog. Neurobiol..

[32] Kim SK, Tsao DD, Suh GSB, Miguel-Aliaga I (2021). Discovering signaling mechanisms governing metabolism and metabolic diseases with *Drosophila*. Cell Metab..

[33] Huang R (2020). High-fat diet enhances starvation-induced hyperactivity via sensitizing hunger-sensing neurons in *Drosophila*. Elife.

[34] Pool AH, Scott K (2014). Feeding regulation in *Drosophila*. Curr. Opin. Neurobiol..

[35] Qi W, Wang G, Wang L (2021). A novel satiety sensor detects circulating glucose and suppresses food consumption via insulin-producing cells in *Drosophila*. Cell Res..

[36] Yang Z (2018). A post-ingestive amino acid sensor promotes food consumption in *Drosophila*. Cell Res..

[37] Melcher C, Pankratz MJ (2005). Candidate gustatory interneurons modulating feeding behavior in the *Drosophila* brain. PLoS Biol..

[38] Sun J (2017). *Drosophila* FIT is a protein-specific satiety hormone essential for feeding control. Nat. Commun..

[39] Martelli C (2017). SIFamide translates hunger signals into appetitive and feeding behavior in *Drosophila*. Cell Rep..

[40] Hergarden AC, Tayler TD, Anderson DJ (2012). Allatostatin-A neurons inhibit feeding behavior in adult *Drosophila*. Proc. Natl. Acad. Sci. USA.

[41] Min S (2016). Identification of a peptidergic pathway critical to satiety responses in *Drosophila*. Curr. Biol..

[42] Oh Y (2019). A glucose-sensing neuron pair regulates insulin and glucagon in *Drosophila*. Nature.

[43] Hentze JL, Carlsson MA, Kondo S, Nassel DR, Rewitz KF (2015). The neuropeptide allatostatin A regulates metabolism and feeding decisions in *Drosophila*. Sci. Rep..

[44] Antonio J (2020). Effects of dietary protein on body composition in exercising individuals. Nutrients.

[45] Journel M, Chaumontet C, Darcel N, Fromentin G, Tome D (2012). Brain responses to high-protein diets. Adv. Nutr..

[46] Jouandin P (2022). Lysosomal cystine mobilization shapes the response of TORC1 and tissue growth to fasting. Science.

[47] Zhang D (2022). Important hormones regulating lipid metabolism. Molecules.

[48] Toprak U (2020). The role of peptide hormones in insect lipid metabolism. Front. Physiol..

[49] Masuyama K, Zhang Y, Rao Y, Wang JW (2012). Mapping neural circuits with activity-dependent nuclear import of a transcription factor. J. Neurogenet..

[50] Kendroud S (2018). Structure and development of the subesophageal zone of the *Drosophila* brain. II. Sensory compartments. J. Comp. Neurol..

[51] Allen AM (2020). A single-cell transcriptomic atlas of the adult *Drosophila* ventral nerve cord. Elife.

[52] Zhao J, Wu Y, Rong X, Zheng C, Guo J (2020). Anti-lipolysis induced by insulin in diverse pathophysiologic conditions of adipose tissue. Diabetes Metab. Syndr. Obes..

[53] Zhang YV, Aikin TJ, Li Z, Montell C (2016). The basis of food texture sensation in *Drosophila*. Neuron.

[54] Shearin HK, Quinn CD, Mackin RD, Macdonald IS, Stowers RS (2018). t-GRASP, a targeted GRASP for assessing neuronal connectivity. J. Neurosci. Methods.

[55] Klapoetke NC (2014). Independent optical excitation of distinct neural populations. Nat. Methods.

[56] Sunter D, Hewson AK, Lynam S, Dickson SL (2001). Intracerebroventricular injection of neuropeptide FF, an opioid modulating neuropeptide, acutely reduces food intake and stimulates water intake in the rat. Neurosci. Lett..

[57] Dockray GJ (2004). The expanding family of -RFamide peptides and their effects on feeding behaviour. Exp. Physiol..

[58] Templeman I (2021). A randomized controlled trial to isolate the effects of fasting and energy restriction on weight loss and metabolic health in lean adults. Sci. Transl. Med..

[59] Ravi P, Trivedi D, Hasan G (2018). FMRFa receptor stimulated Ca2+ signals alter the activity of flight modulating central dopaminergic neurons in *Drosophila melanogaster*. PLoS Genet..

[60] Klose MK, Dason JS, Atwood HL, Boulianne GL, Mercier AJ (2010). Peptide-induced modulation of synaptic transmission and escape response in *Drosophila* requires two G-protein-coupled receptors. J. Neurosci..

[61] Lenz O, Xiong J, Nelson MD, Raizen DM, Williams JA (2015). FMRFamide signaling promotes stress-induced sleep in *Drosophila*. Brain Behav. Immun..

[62] Clark J, Milakovic M, Cull A, Klose MK, Mercier AJ (2008). Evidence for postsynaptic modulation of muscle contraction by a *Drosophila* neuropeptide. Peptides.

[63] Sandvik GK, Hodne K, Haug TM, Okubo K, Weltzien FA (2014). RFamide peptides in early vertebrate development. Front. Endocrinol..

[64] Nguyen T, Marusich J, Li JX, Zhang Y (2020). Neuropeptide FF and its receptors: therapeutic applications and ligand development. J. Med. Chem..

[65] Sveidahl Johansen O (2021). Lipolysis drives expression of the constitutively active receptor GPR3 to induce adipose thermogenesis. Cell.

[66] Akbari P (2021). Sequencing of 640,000 exomes identifies GPR75 variants associated with protection from obesity. Science.

[67] Ahima RS, Antwi DA (2008). Brain regulation of appetite and satiety. Endocrinol. Metab. Clin. North Am..

[68] Jourjine N, Mullaney BC, Mann K, Scott K (2016). Coupled sensing of hunger and thirst signals balances sugar and water consumption. Cell.

[69] Chopra G, Kaushik S, Kain P (2022). Nutrient sensing via gut in *Drosophila melanogaster*. Int. J. Mol. Sci..

[70] Ko KI (2015). Starvation promotes concerted modulation of appetitive olfactory behavior via parallel neuromodulatory circuits. Elife.

[71] Nassel DR, Zandawala M (2022). Endocrine cybernetics: neuropeptides as molecular switches in behavioural decisions. Open Biol..

[72] Guo D (2021). Cholecystokinin-like peptide mediates satiety by inhibiting sugar attraction. PLoS Genet..

[73] Yoshinari Y (2021). The sugar-responsive enteroendocrine neuropeptide F regulates lipid metabolism through glucagon-like and insulin-like hormones in *Drosophila melanogaster*. Nat. Commun..

[74] Hong SH (2012). Minibrain/Dyrk1a regulates food intake through the Sir2-FOXO-sNPF/NPY pathway in *Drosophila* and mammals. PLoS Genet..

[75] Deng B (2019). Chemoconnectomics: mapping chemical transmission in *Drosophila*. Neuron.

[76] Qi W (2015). A quantitative feeding assay in adult *Drosophila* reveals rapid modulation of food ingestion by its nutritional value. Mol. Brain.

[77] Yu Y (2016). Regulation of starvation-induced hyperactivity by insulin and glucagon signaling in adult. Drosophila. Elife.

[78] Takeuchi K (2009). Changes in temperature preferences and energy homeostasis in dystroglycan mutants. Science.

