# Supplementary material for: Dietary cysteine drives body fat loss via FMRFamide signaling in Drosophila and mouse
Source: Cell Res. 2023 Apr 13;33(6):434–47. doi: 10.1038/s41422-023-00800-8 (PMC10235132; doi:10.1038/s41422-023-00800-8)
Supplement: Supplementary file 1 — Supplementary information, Fig. S1 [file 41422_2023_800_MOESM1_ESM.pdf]

Fig. S1

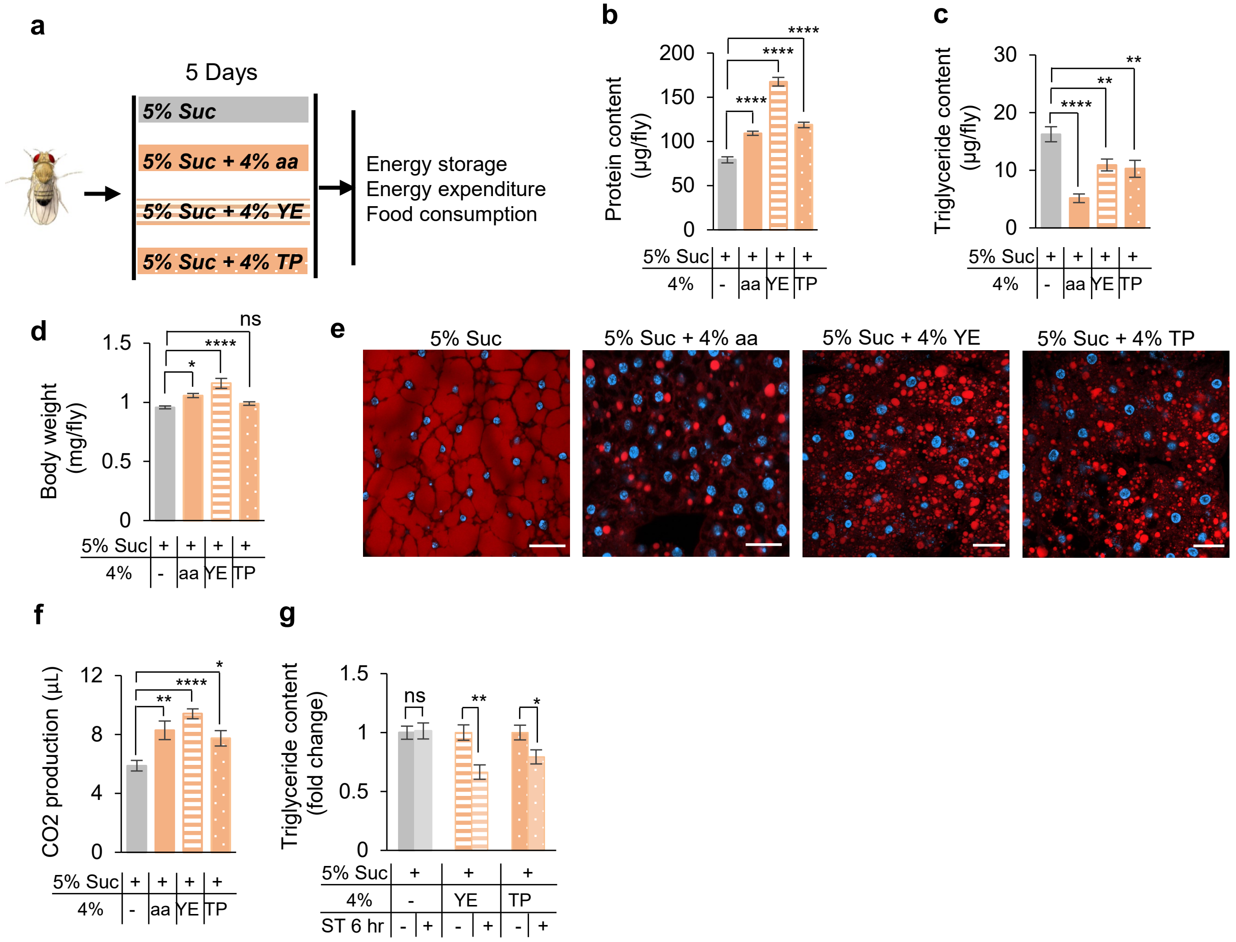

Fig. S1: Excess protein added to 5% sucrose also exerted fat loss effect in fruit flies.

a The illustration of experimental design in Figs. S1b-g**.** Wild-type *Oregon-R* virgin female flies were collected and fed with 5% sucrose with (orange) or without (grey) indicated proteins (4%) for 5 days and then subjected to different metabolic and behavioral assays. (**b-d**) The content of different nutrient types after HPD treatment, including protein (b, n=10), triglyceride (c, n=8-16), and body weight (d, n=5) of indicated treatment groups. **e** Nile Red staining of the fat bodies collected from the indicated treatment groups, lipids in red and nucleus in blue. Scale bar, 20 µm. **f** CO_2 production of indicated treatment groups in 1 hour (n=9-11 biological replicates, each containing 5 flies). **g** Triglyceride content of flies pre-fed with different HPD for 5 days and then starved for 6 hours or not (n=23-32). ns, $p > 0.05$; *$p < 0.05$; **$p < 0.01$; ****$p < 0.0001$. One-way ANOVA followed by post hoc test with Bonferroni correction was used for multiple comparisons when applicable.
