# Supplementary material for: Dietary cysteine drives body fat loss via FMRFamide signaling in Drosophila and mouse
Source: Cell Res. 2023 Apr 13;33(6):434–47. doi: 10.1038/s41422-023-00800-8 (PMC10235132; doi:10.1038/s41422-023-00800-8)
Supplement: Supplementary file 2 — Supplementary information, Fig. S2 [file 41422_2023_800_MOESM2_ESM.pdf]

Fig. S2

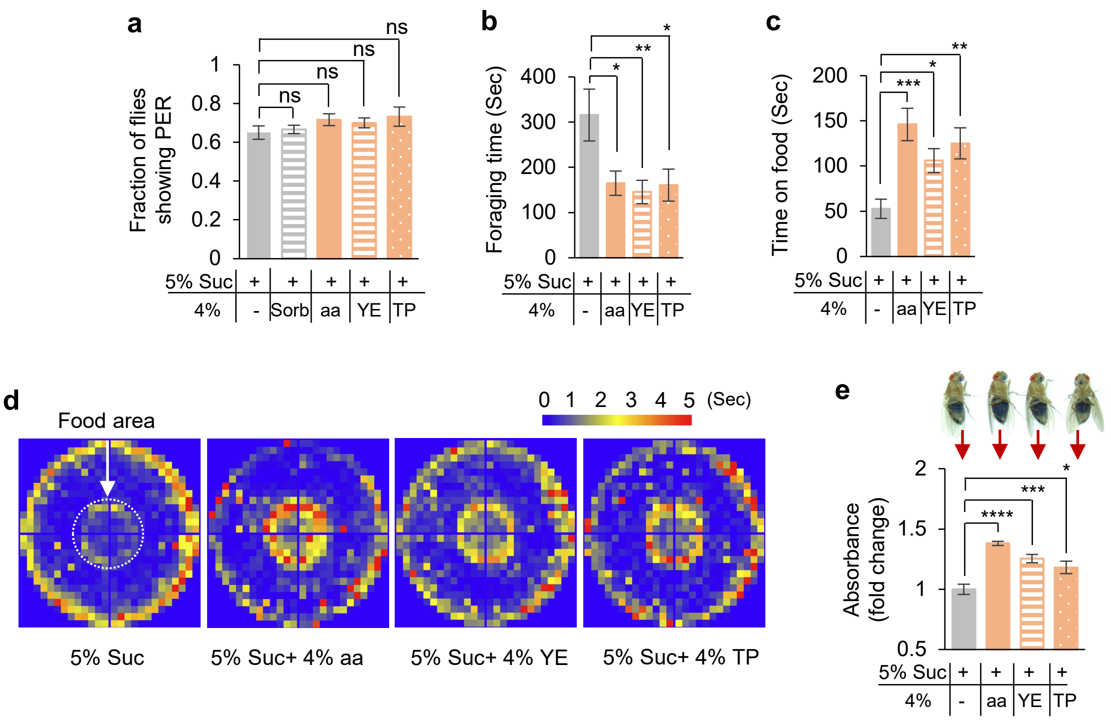

Fig. S2: HPD enhanced foraging behavior in flies.

a Fraction of pre-starved (for 24 hours) flies showing PER responses to the indicated food types (n=6, groups each of 10 flies). (**b-d**) Flies pre-fed with different types of HPD in the video recording-based food seeking assay. HPD-fed flies were starved for 6 hours and then transferred into a behavioral chamber with a small food patch (5% sucrose) in the center, and their positions were recorded by a camera placed on top and analyzed by a custom computer program (n=41-59). **b** Latency to reach the food source. **c** Total duration on food. **d** Represent spatial distribution during the 10-minute assay. **e** Food consumption of flies. Flies were pre-fed with different types of HPD for 5 days, starved for 12 hours, and then presented with 5% sucrose (containing 0.5% Brilliant Blue) for 10 min (n=6-13). *$p < 0.05$; **$p < 0.01$; ***$p < 0.001$; ****$p < 0.0001$. One-way followed by post hoc test with Bonferroni correction was used for multiple comparisons when applicable.
