# Supplementary material for: Dietary cysteine drives body fat loss via FMRFamide signaling in Drosophila and mouse
Source: Cell Res. 2023 Apr 13;33(6):434–47. doi: 10.1038/s41422-023-00800-8 (PMC10235132; doi:10.1038/s41422-023-00800-8)
Supplement: Supplementary file 3 — Supplementary information, Fig. S3 [file 41422_2023_800_MOESM3_ESM.pdf]

Fig. S3

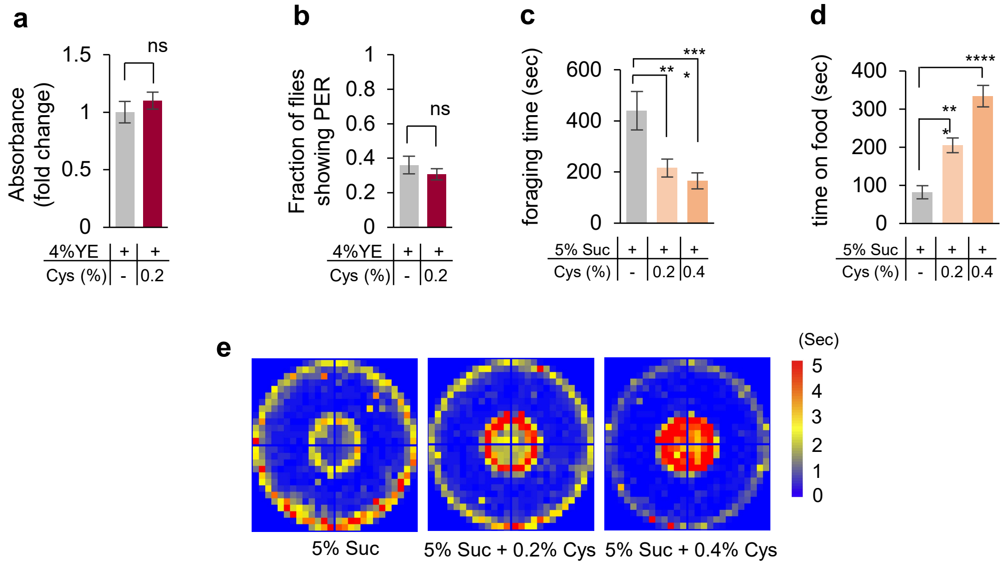

Fig. S3: Dietary cysteine enhanced foraging behavior in flies.

a Food consumption of flies fed with 4% YE (grey) or 4% YE added with 0.2% cysteine (red) for 5 days (n=7-8). **b** Fraction of flies showing PER response to 4% YE added with 0.2% cysteine (n=5 groups, each of 10 flies). (**c-e**) Flies pre-fed with dietary cysteine in the video recording-based food seeking assay. Cysteine-fed flies were starved for 6 hours and then transferred into a behavioral chamber with a small food patch (5% sucrose) in the center, and their positions were recorded by a camera placed on top and analyzed by a custom computer program (n=31-43). **c** Latency to reach the food source. **d** Total duration on food. **e** Represent spatial distribution during the 10-minute assay. **$p < 0.01$; ***$p < 0.001$; ****$p < 0.0001$. One-way followed by post hoc test with Bonferroni correction was used for multiple comparisons when applicable.
