# Supplementary material for: Dietary cysteine drives body fat loss via FMRFamide signaling in Drosophila and mouse
Source: Cell Res. 2023 Apr 13;33(6):434–47. doi: 10.1038/s41422-023-00800-8 (PMC10235132; doi:10.1038/s41422-023-00800-8)
Supplement: Supplementary file 4 — Supplementary information, Fig. S4 [file 41422_2023_800_MOESM4_ESM.pdf]

Fig. S4

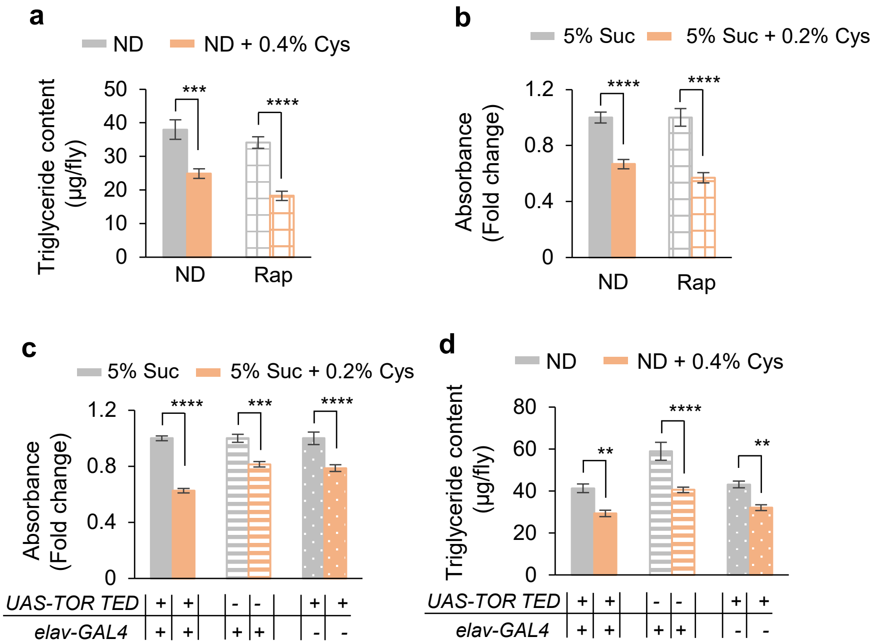

Fig. S4: TOR signaling was not related to cysteine-induced fat loss and feeding suppression.

a Triglyceride content of rapamycin (400 μM)-treated flies with or without cysteine for 3 days (n=6). **b** Consumption of 5% sucrose or 5% sucrose added with cysteine of indicated flies (n=6). **c** Consumption of 5% sucrose or 5% sucrose added with cysteine of flies of the indicated genotypes (n=6). **d** Triglyceride content (n=6-8) of flies of the indicated genotypes fed with ND or ND added with 0.2% cysteine for 5 days. **p < 0.01; ***p < 0.001; ****p < 0.0001. One-way followed by post hoc test with Bonferroni correction was used for multiple comparisons when applicable.
