# Supplementary material for: Dietary cysteine drives body fat loss via FMRFamide signaling in Drosophila and mouse
Source: Cell Res. 2023 Apr 13;33(6):434–47. doi: 10.1038/s41422-023-00800-8 (PMC10235132; doi:10.1038/s41422-023-00800-8)
Supplement: Supplementary file 5 — Supplementary information, Fig. S5 [file 41422_2023_800_MOESM5_ESM.pdf]

Fig. S5

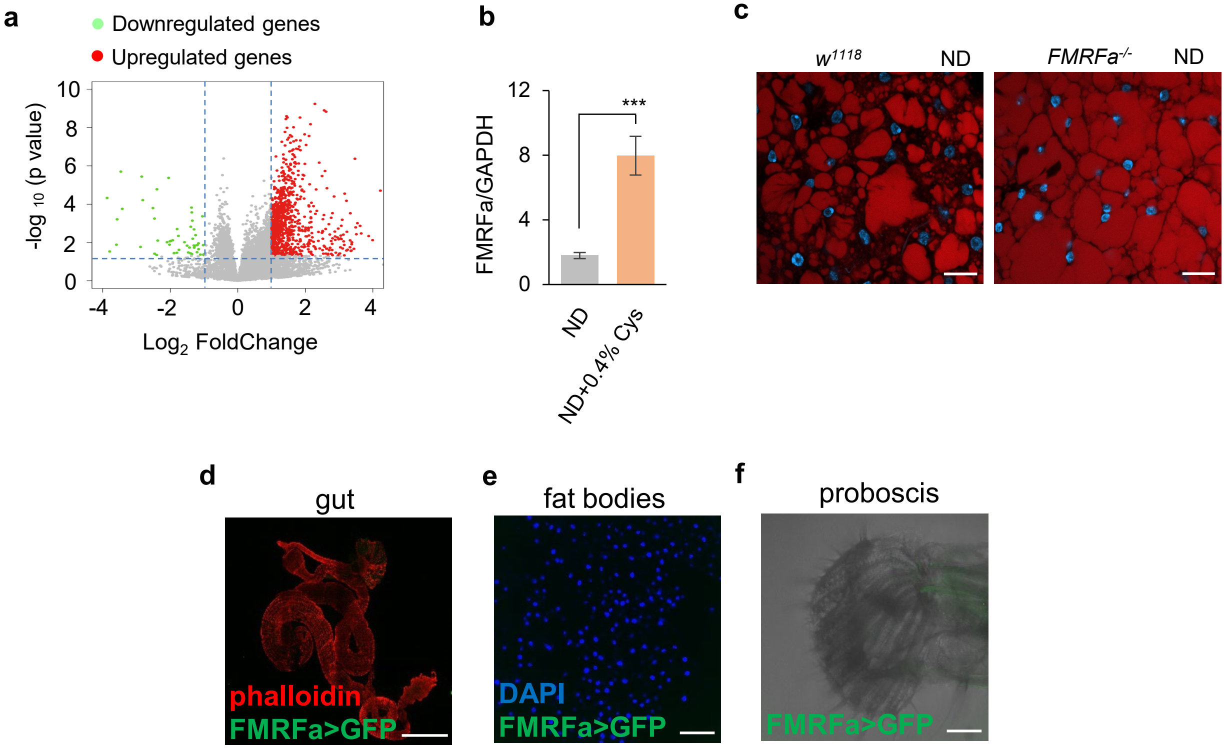

Fig. S5: FMRFa signaling suppressed fat storage under ND feeding.

a RNAseq analysis of flies fed with 5% sucrose or 5% sucrose added with 0.2% cysteine for 5 days (n=6). Volcano plot showed differentially expressed genes between the two groups (green: downregulated genes in the cysteine group; red: upregulated genes in the cysteine group). The horizontal line indicated the significance threshold (p = 0.05), and the vertical lines indicated two-fold change threshold. **b** *FMRFa* mRNA levels of flies fed with ND or ND plus cysteine by qPCR (n=6). **c** Nile Red staining of the fat bodies collected from the indicated genotypes, lipids in red and nucleus in blue. Scale bar, 20 μm. (**d-f**) FMRFa expression in the gut (d, Scale bar, 500 μm), the fat bodies (e, Scale bar, 50 μm), and the proboscis (f, Scale bar, 50 μm), illustrated by mCD8::GFP driven by *FMRFa*^{GAL4}. ***p < 0.001. Student's t-test was used for unpaired -wise comparisons.
