# Supplementary material for: Dietary cysteine drives body fat loss via FMRFamide signaling in Drosophila and mouse
Source: Cell Res. 2023 Apr 13;33(6):434–47. doi: 10.1038/s41422-023-00800-8 (PMC10235132; doi:10.1038/s41422-023-00800-8)
Supplement: Supplementary file 6 — Supplementary information, Fig. S6 [file 41422_2023_800_MOESM6_ESM.pdf]

Fig. S6

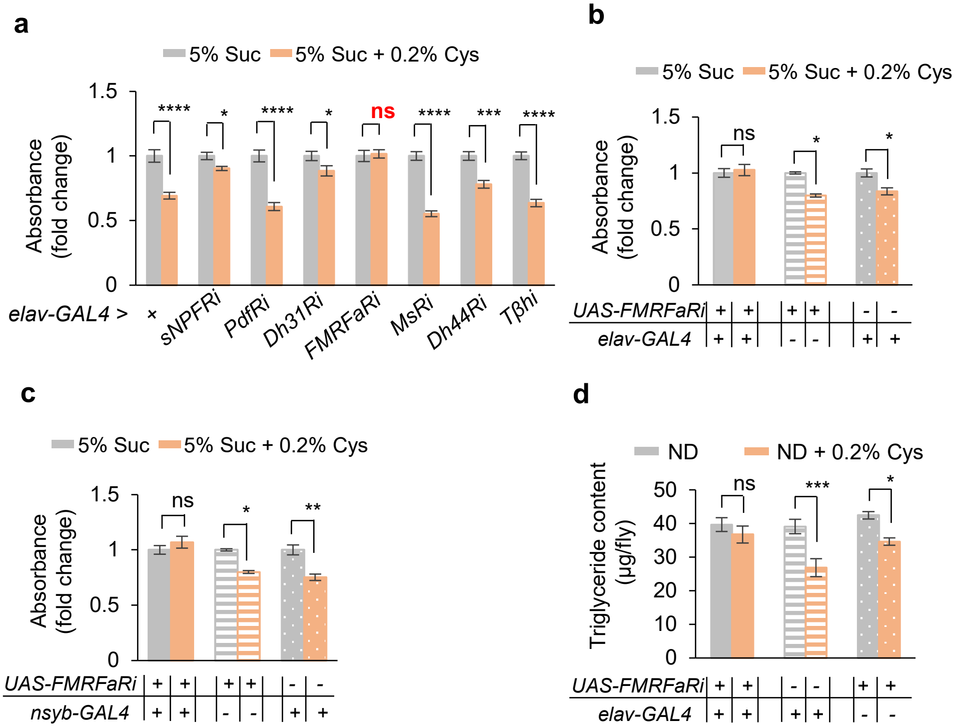

Fig. S6: Neuronal FMRFaR mediated the inhibitory effect of cysteine on feeding behavior.

(**a-c**) Consumption of 5% sucrose or 5% sucrose added with cysteine of flies of the indicated genotypes (n=5-9). **d** Triglyceride content of flies of the indicated genotypes fed with ND or ND added with 0.2% cysteine for 5 days (n=7-8). ns, $p > 0.05$; *$p < 0.05$; **$p < 0.01$; ***$p < 0.001$; ****$p < 0.0001$. One-way ANOVA followed by post hoc test with Bonferroni correction were used for multiple comparisons when applicable.
