# Supplementary material for: Dietary cysteine drives body fat loss via FMRFamide signaling in Drosophila and mouse
Source: Cell Res. 2023 Apr 13;33(6):434–47. doi: 10.1038/s41422-023-00800-8 (PMC10235132; doi:10.1038/s41422-023-00800-8)
Supplement: Supplementary file 7 — Supplementary information, Fig. S7 [file 41422_2023_800_MOESM7_ESM.pdf]

Fig. S7

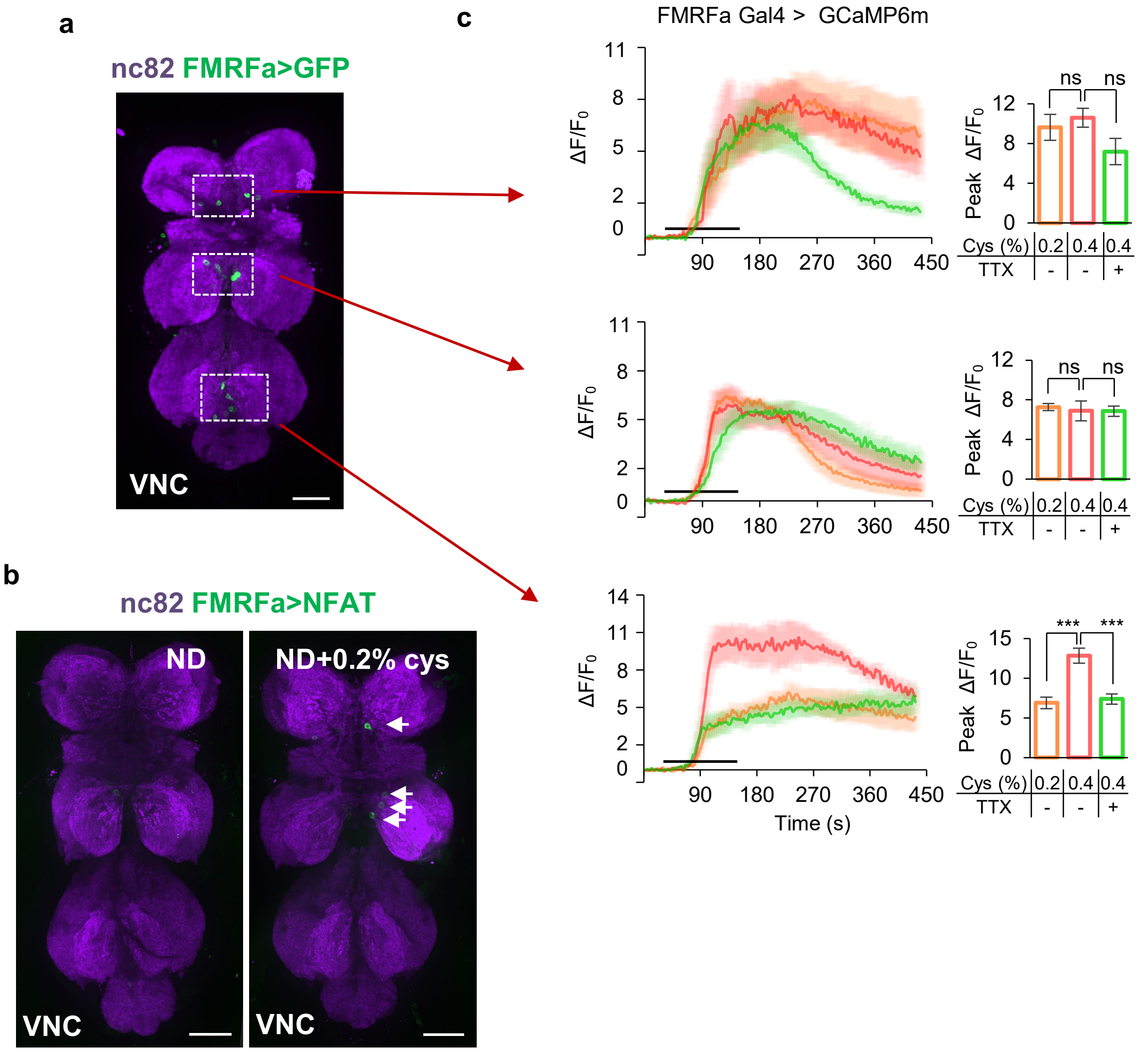

Fig. S7: FMRFa⁺ neurons in the VNC were directly activated by cysteine.

a FMRFa expression in the VNC, illustrated by mCD8::GFP driven by $FMRFa^{GAL4}$. Scale bar, 50 μm. **b** The activity of FMRFa⁺ neurons in VNC after cysteine feeding measured by NFAT system. Scale bar, 50 μm. **c** Representative traces and quantification of ex vivo calcium responses of FMRFa⁺ neurons during perfusion of cysteine with or without TTX (n=6). ns, $p > 0.05$; ***$p < 0.001$. One-way and two-way ANOVA followed by post hoc test with Bonferroni correction were used for multiple comparisons when applicable.
