# Supplementary material for: Dietary cysteine drives body fat loss via FMRFamide signaling in Drosophila and mouse
Source: Cell Res. 2023 Apr 13;33(6):434–47. doi: 10.1038/s41422-023-00800-8 (PMC10235132; doi:10.1038/s41422-023-00800-8)
Supplement: Supplementary file 8 — Supplementary information, Fig. S8 [file 41422_2023_800_MOESM8_ESM.pdf]

Fig. S8

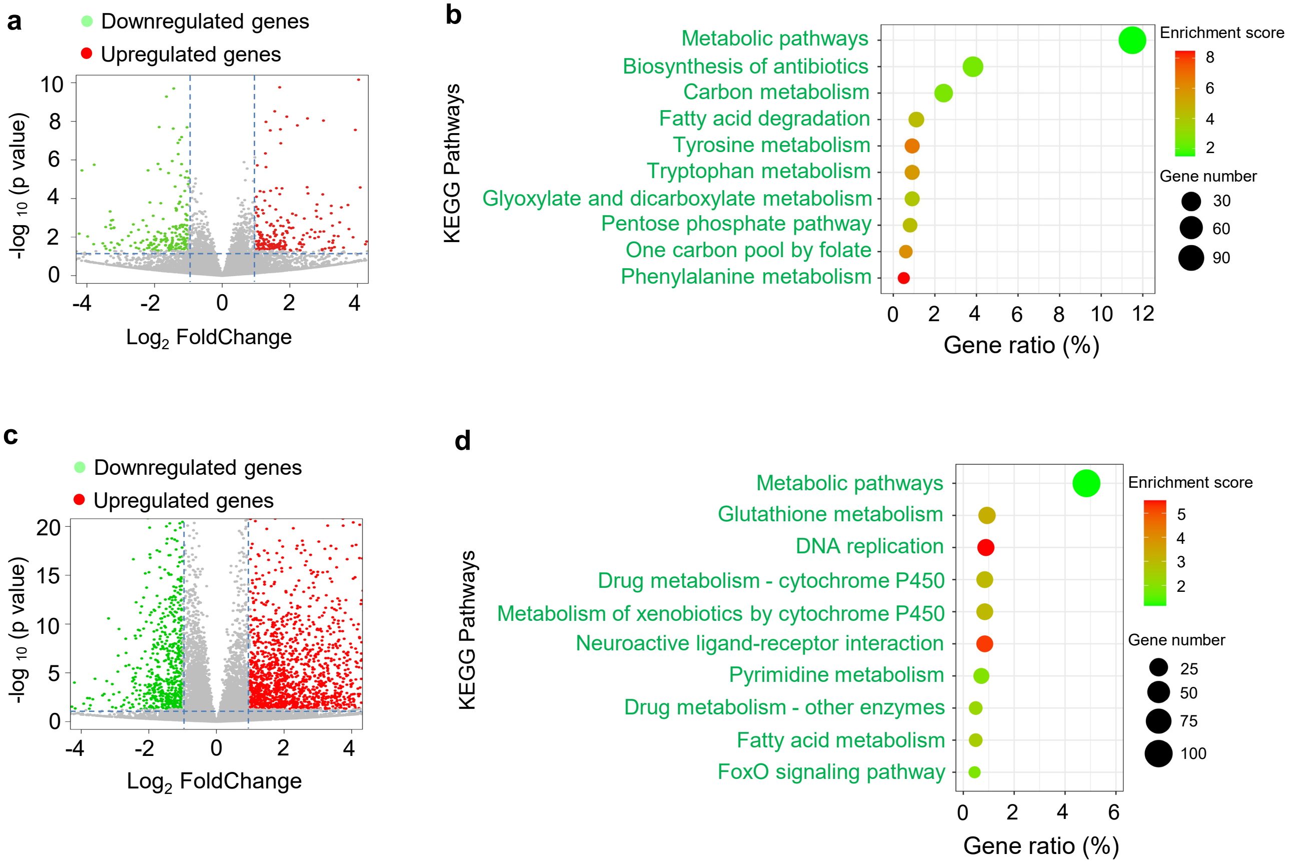

Fig. S8: Analysis of gene expression changes in the fat body induced by dietary cysteine and FMRFa injection.

(**a-b**) RNAseq analysis of the fat body of flies fed with 5% sucrose or 5% sucrose added with 0.2% cysteine for 5 days (n=6). **a** Volcano plot showed differentially expressed genes between the two groups (green: downregulated genes in the cysteine group; red: upregulated genes in the cysteine group). The horizontal line indicated the significance threshold (p = 0.05), and the vertical lines indicated two-fold change threshold. **b** The top influenced signaling pathway by dietary cysteine from KEGG analysis. (**c-d**) RNAseq analysis of the fat body of flies incubated with synthetic FMRFa (20 μg/ml) or not (n=5-6). Volcano plot was showed in c and KEGG analysiswas shown in d.
