# Supplementary material for: Dietary cysteine drives body fat loss via FMRFamide signaling in Drosophila and mouse
Source: Cell Res. 2023 Apr 13;33(6):434–47. doi: 10.1038/s41422-023-00800-8 (PMC10235132; doi:10.1038/s41422-023-00800-8)
Supplement: Supplementary file 9 — Supplementary information, Fig. S9 [file 41422_2023_800_MOESM9_ESM.pdf]

Fig. S9

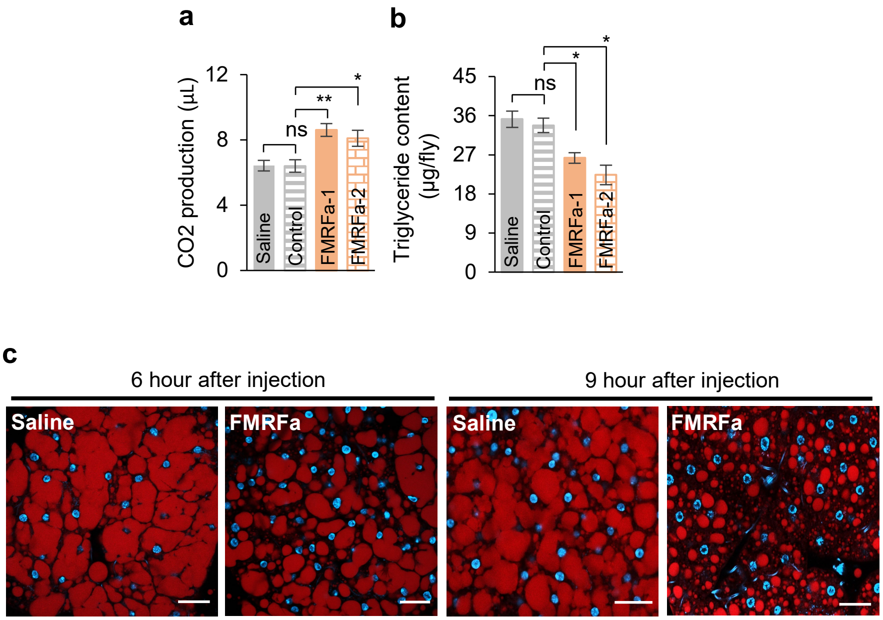

Fig. S9: Injection of synthetic FMRFa peptides induced fat loss.

(**a-b**) 1 hour CO_2 production (a, n=9-19) and triglyceride content (b, n=6-8) of flies injected with saline, controlled peptide or synthetic FMRFa (FMRFa-1 and FMRFa-2). Triglyceride content was assayed 6 hours after the microinjection. **c** Nile Red staining of the fat bodies of flies after saline or FMRFa microinjection for 6 or 9 hours. Scale bar, 20 µm. ns, $p > 0.05$; *$p < 0.05$; **$p < 0.01$. One-way ANOVA followed by post hoc test with Bonferroni correction were used for multiple comparisons when applicable.
