# Supplementary material for: Dietary cysteine drives body fat loss via FMRFamide signaling in Drosophila and mouse
Source: Cell Res. 2023 Apr 13;33(6):434–47. doi: 10.1038/s41422-023-00800-8 (PMC10235132; doi:10.1038/s41422-023-00800-8)
Supplement: Supplementary file 10 — Supplementary information, Fig. S10 [file 41422_2023_800_MOESM10_ESM.pdf]

Fig. S10

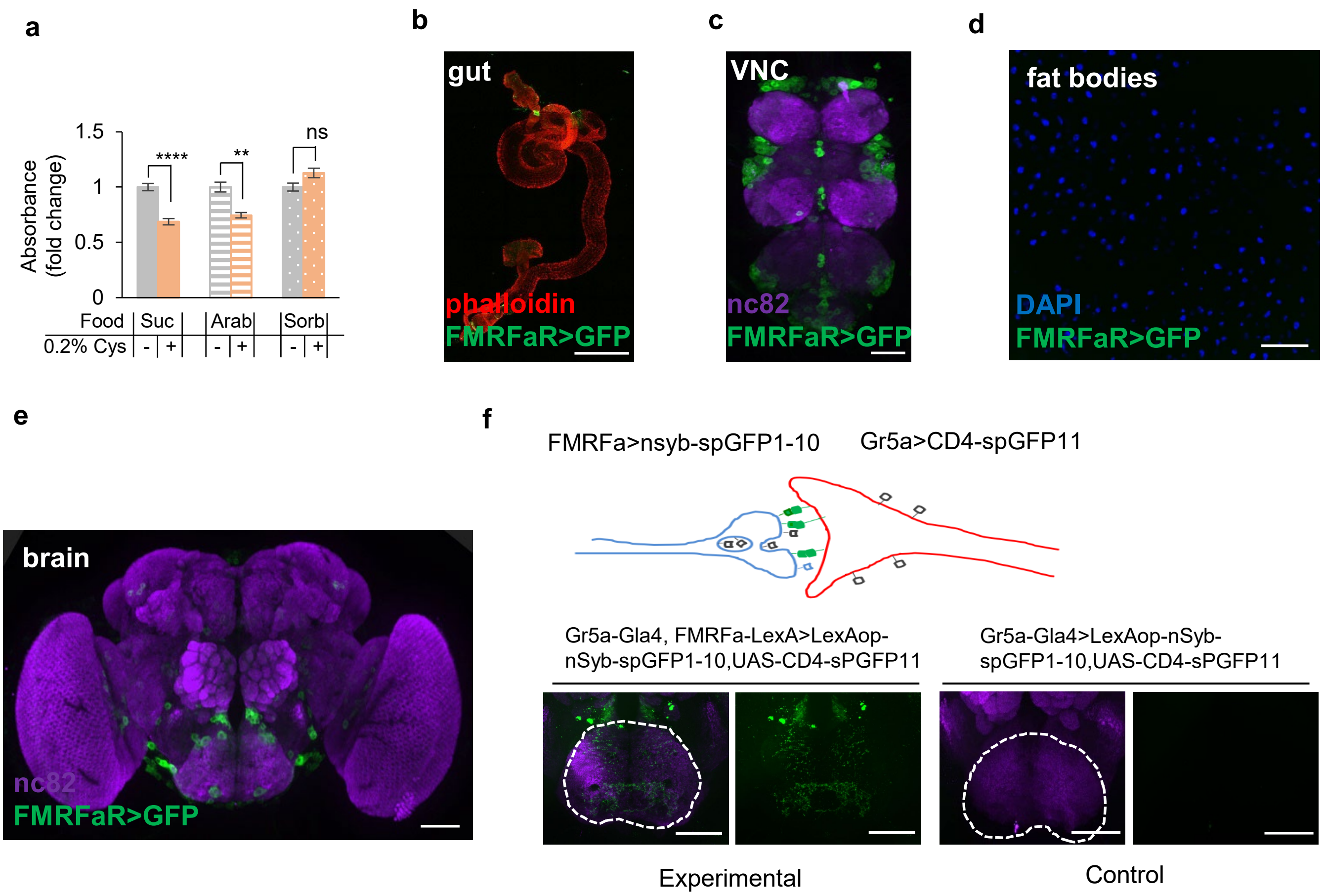

Fig. S10: FMRFaR expression in different tissues.

a Food consumption of pre-starved flies assayed with the indicated sugar types with or without 0.2% cysteine (n=6-12). (**b-e**) FMRFaR expression in the gut (b, Scale bar, 500 μm), the VNC (c, Scale bar, 50 μm), the fat bodies (d, Scale bar, 50 μm), and the brain (e, Scale bar, 50 μm), illustrated by mCD8::GFP driven by *FMRFa*^{GAL4}. **f** (top) Schematic diagram of nSyb-GRASP between FMRFa^{+} and Gr5a^{+} neurons. (left) nSyb-GRASP signals between FMRFa^{+} neurons and Gr5a^{+} neurons in the SEZ region (dotted line). (right) In the control groups, no nSyb-GRASP signal was seen. Scale bar represents 50 μm.
