# Supplementary material for: Dietary cysteine drives body fat loss via FMRFamide signaling in Drosophila and mouse
Source: Cell Res. 2023 Apr 13;33(6):434–47. doi: 10.1038/s41422-023-00800-8 (PMC10235132; doi:10.1038/s41422-023-00800-8)
Supplement: Supplementary file 11 — Supplementary information, Fig. S11 [file 41422_2023_800_MOESM11_ESM.pdf]

Fig. S11

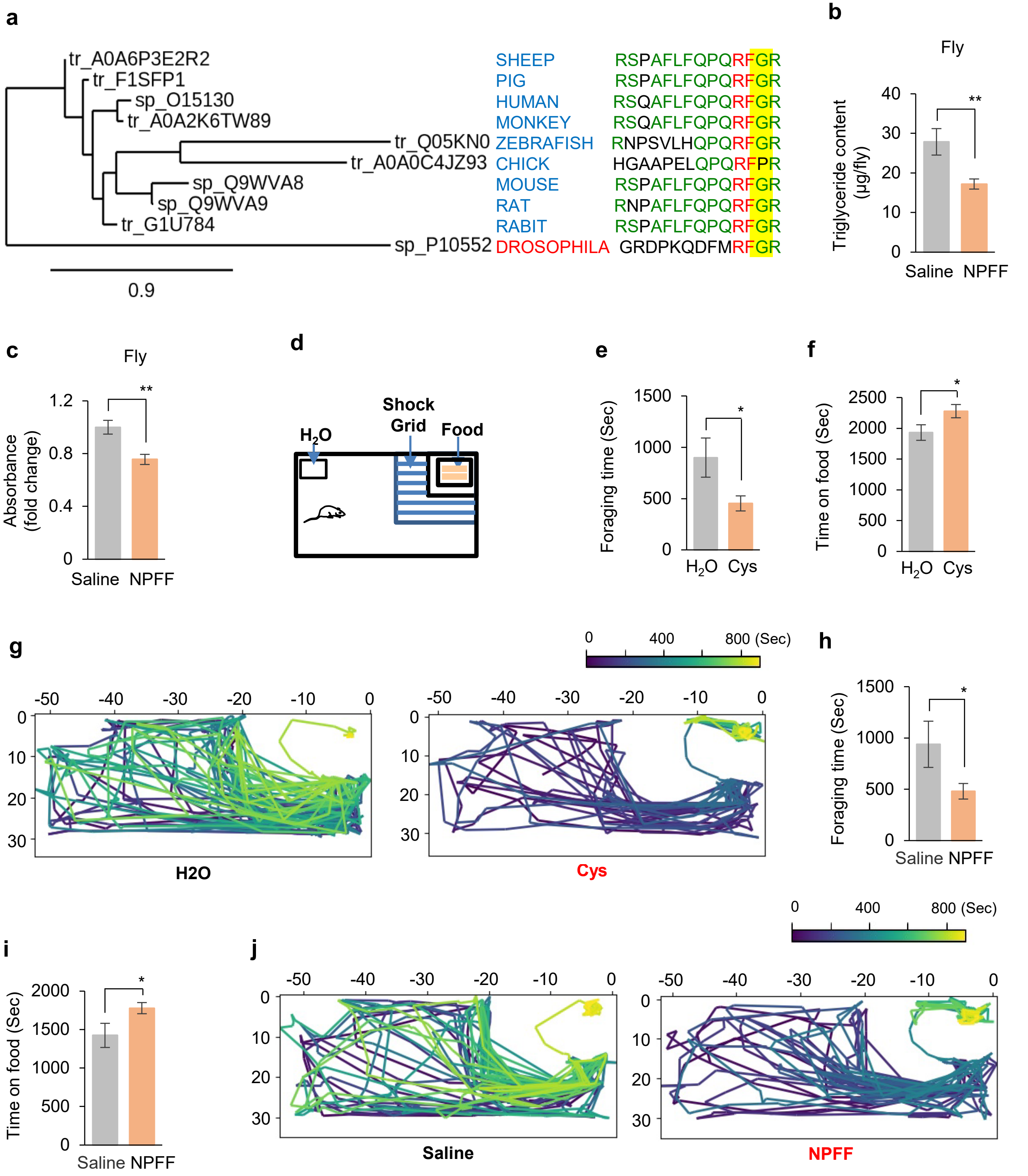
a
tr_A0A6P3E2R2
tr_F1SFP1
sp_O15130
tr_A0A2K6TW89
tr_Q05KN0
tr_A0A0C4JZ93
sp_Q9WVA8
sp_Q9WVA9
tr_G1U784
sp_P10552
0.9
SHEEP RSPAFLFQPQRFGR
PIG RSPAFLFQPQRFGR
HUMAN RSQAFLFQPQRFGR
MONKEY RSQAFLFQPQRFGR
ZEBRAFISH RNPSVLHQPQRFGR
CHICK HGAAPELQPQRFPR
MOUSE RSPAFLFQPQRFGR
RAT RNPAFLFQPQRFGR
RABIT RSPAFLFQPQRFGR
DROSOPHILA GRDPKQDFMRFGR
b
Fly
Triglyceride content (μg/fly)
Saline NPFF
c
Fly
Absorbance (fold change)
Saline NPFF
d
H2O
Shock Grid
Food
e
Foraging time (Sec)
H2O Cys
f
Time on food (Sec)
H2O Cys
g
0 400 800 (Sec)
H2O
Cys
h
Foraging time (Sec)
Saline NPFF
i
Time on food (Sec)
Saline NPFF
j
0 400 800 (Sec)
Saline
NPFF

Fig. S11: Dietary cysteine and NPFF administration enhanced foraging behavior in mice.

a Comparison of the core amino acid sequence of *Drosophila* FMRFamide with various vertebrate analogous genes. (**b-c**) Triglyceride content (b) and food consumption (c) of flies injected with synthetic NPFF (n=8-9). Individual flies were injected twice in 6 hours before measurement of triglyceride content, and injected once 30 mins before the feeding assay. **d** Schematic illustration of the video recording-based foraging assay for mice. (**e-f**) Latency to reach the food source (e) and total duration on food (f) for mice orally garaged with cysteine (4 mmol/kg per day for 60 days) (n=11-14). Mice were starved for 24 hours before the foraging assays. **g** The pseudocolored (as a function of time) moving trajectories of individual mice. (**h-i**) Latency to reach the food source (h) and total duration on food (i) for mice with IV administration of NPFF (3.2 mg/kg per 12 hours for 40 days) (n=28-29). Mice were starved for 24 hours before the foraging assays. **j** The pseudocolored (as a function of time) moving trajectories of individual mice. *$p < 0.05$; **$p < 0.01$. Student's t-test was used for pair-wise comparisons.
