# Supplementary material for: Dietary cysteine drives body fat loss via FMRFamide signaling in Drosophila and mouse
Source: Cell Res. 2023 Apr 13;33(6):434–47. doi: 10.1038/s41422-023-00800-8 (PMC10235132; doi:10.1038/s41422-023-00800-8)
Supplement: Supplementary file 12 — Supplementary information, Fig. S12 [file 41422_2023_800_MOESM12_ESM.pdf]

Fig. S12

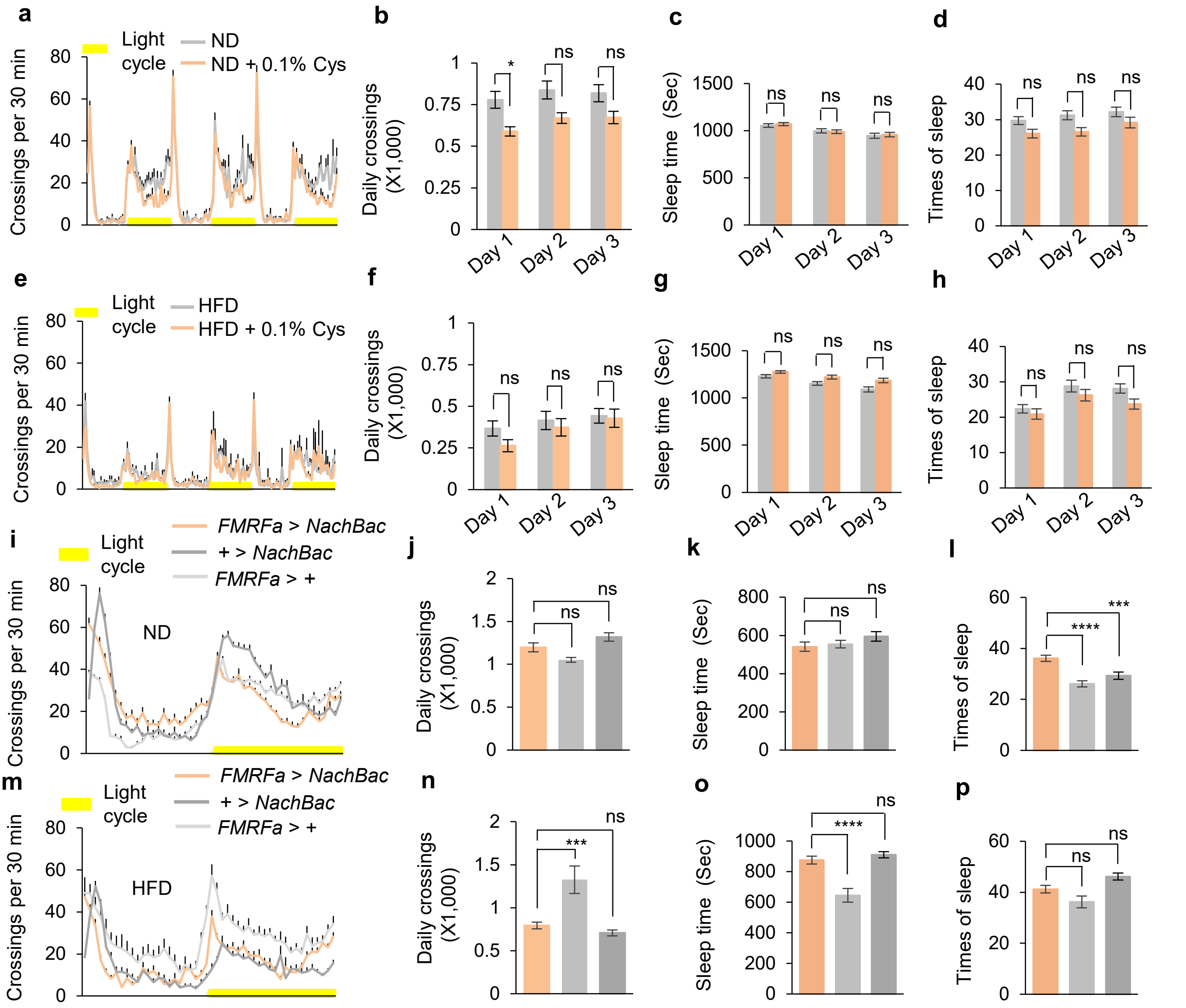

Fig. S12. Dietary cysteine and FMRFa signaling did not affect locomotion and sleep in flies.

(**a-b,e-f,i-j,m-n**) Average daily midline crossing activity of flies of the indicated genotypes and food supplement using the DAMS-based locomotion assay (n=44-48). (**c,g,k,o**) Daily sleep time of flies of the indicated genotypes and food supplement using the DAMS-based locomotion assay (n=44-48). (**d,h,l,p**) Daily number of sleep bouts of flies of the indicated genotypes and food supplement using the DAMS-based locomotion assay (n=44-48). ns, $p > 0.05$; *$p < 0.05$; ***$p < 0.001$; ****$p < 0.0001$. One-way ANOVA followed by post hoc test with Bonferroni correction was used for multiple comparisons when applicable.
